# Supplementary material for: Evaluating 3D-printed models of coronary anomalies: a survey among clinicians and researchers at a university hospital in the UK
Source: BMJ Open. 2019 Mar 8;9(3):e025227. doi: 10.1136/bmjopen-2018-025227 (PMC6430025; doi:10.1136/bmjopen-2018-025227)
Supplement: Author's manuscript [file bmjopen-2018-025227.draft_revisions.pdf]

BMJ Open

BMJ Open is committed to open peer review. As part of this commitment we make the peer review history of every article we publish publicly available.

When an article is published we post the peer reviewers' comments and the authors' responses online. We also post the versions of the paper that were used during peer review. These are the versions that the peer review comments apply to.

The versions of the paper that follow are the versions that were submitted during the peer review process. They are not the versions of record or the final published versions. They should not be cited or distributed as the published version of this manuscript.

BMJ Open is an open access journal and the full, final, typeset and author-corrected version of record of the manuscript is available on our site with no access controls, subscription charges or pay-per-view fees (http://bmjopen.bmj.com).

If you have any questions on BMJ Open's open peer review process please email info.bmjopen@bmj.com

BMJ Open

Revealing the anatomical complexity of coronary anomalies: 3D printing coronary models from computed tomography data

Journal:	*BMJ Open*
Manuscript ID	bmjopen-2018-025227
Article Type:	Research
Date Submitted by the Author:	06-Jul-2018
Complete List of Authors:	Lee, Matthew ; University of Bristol School of Clinical Science<br>Moharem-Elgamal, Sarah; University of Bristol, Bristol Heart Institute<br>Beckingham, Rylan; University of Bristol<br>Hamilton, Mark; University Hospitals Bristol NHS Foundation Trust<br>Manghat, Nathan; University Hospitals Bristol NHS Foundation Trust<br>Milano, Elena; Universita di Verona<br>Bucciarelli Ducci, Chiara; University of Bristol<br>Caputo, Massimo; University of Bristol, Bristol Heart Institute<br>Biglino, Giovanni; University of Bristol School of Clinical Science, Bristol Heart Institute
Keywords:	Coronary artery anomalies, 3D printing, Computed tomography < RADIOLOGY & IMAGING, coronary arteries

SCHOLARONE™ Manuscripts

For peer review only

Revealing the Anatomical Complexity of Coronary Anomalies: 3D Printing Coronary Models from Computed Tomography Data

Matthew Lee[1], Sarah Moharem-Elgamal[2,3], Rylan Beckingham[3], Mark Hamilton[4], Nathan Manghat[4], Elena Giulia Milano[3,5], Chiara Bucciarelli-Ducci[3], Massimo Caputo[3], and Giovanni Biglino[3,6]

[1]Bristol Medical School, University of Bristol, Bristol, UK ; [2]National Heart Institute, Giza, Egypt; [3]Bristol Heart Institute, University of Bristol, Bristol, UK; [4]Radiology Department, University Hospitals Bristol, NHS Foundation Trust, Bristol, UK; [5]Dipartimento di Cardiologia, Universitá di Verona, Verona, Italy; [6]Great Ormond Street Hospital for Children, NHS Foundation Trust, London, UK

Corresponding author:
Dr Giovanni Biglino
Bristol Heart Institute
Bristol Royal Infirmary
Upper Maudlin Street
BS2 8HW Bristol
United Kingdom
Email: g.biglino@bristol.ac.uk
Tel: (44) 117 342 3287

Word count (main text including figure legends): 3,568

Total number of tables: 5

Total number of figures: 3

For peer review only - http://bmjopen.bmj.com/site/about/guidelines.xhtml

Structured Abstract (293 words)

Objective: To evaluate the feasibility of 3D printing models of coronary artery anomalies based on cardiac computed tomography (CT) data and explore their potential for clinical applications.

Design: Cardiac CT datasets of patients with various coronary artery anomalies (n=8) were retrospectively reviewed and processed, reconstructing detailed 3D models to be printed in-house with a desktop 3D printer (Form2, Formlabs) using white resin.

Setting: University Hospital, division of cardiology.

Participants: The CT scans, first, and the then 3D-printed models were presented to groups of clinicians (n=8) and cardiovascular researchers (n=9).

Intervention: Participants were asked to assess different features of the 3D models and to rate the models' overall potential usefulness.

Outcome measures: Models were rated according to clarity of anatomical detail, insight into the coronary abnormality, overall perceived usefulness, and comparison to CT scans. Assessment of model characteristics used Likert-type questions (5-point scale from "Strongly disagree" to "Strongly agree") or a 10-point rating (from 0, lowest, to 10, highest). The questionnaire included a feedback form summarising overall usefulness. Participants' imaging experience (in number of years) was also recorded.

Results: All models were reconstructed and printed successfully, with accurate details showing coronary anatomy (e.g. anomalous coronary artery, coronary roofing, or coronary aneurysm in a patient with Kawasaki syndrome). All clinicians and researchers provided feedback, with both groups finding the models helpful in displaying coronary artery anatomy and abnormalities, and complementary to viewing 3D CT scans. The clinicians' group, who had substantially more imaging expertise, provided more enthusiastic ratings in terms of models' clarity, usefulness, and future use on average.

For peer review only - http://bmjopen.bmj.com/site/about/guidelines.xhtml

Conclusions: 3D printed heart models can be feasibly used to recreate coronary artery anatomy and enhance understanding of coronary abnormalities. Future studies can evaluate their cost-effectiveness, as well as potentially explore other printing techniques and materials.

Key Words

Coronary artery anomalies; 3D printing; Computed Tomography; Coronary arteries

Abbreviations List

3D: Three-dimensional

CT: Computed tomography

CAA: Coronary artery anomalies

ALCAPA: Anomalous left coronary artery from pulmonary artery

TGA: Transposition of the great arteries

Data Statement

Data available upon request.

For peer review only - http://bmjopen.bmj.com/site/about/guidelines.xhtml

For peer review only

Article Summary

Strengths and Limitations of this Study

- The study presents the feasibility of manufacturing 3D models of a range of coronary anomalies, reconstructed from cardiac CT.
- The study gathers evidence toward clinical and research uses of 3D cardiac models, particularly for studying coronary anomalies.
- Clinicians and researchers were involved in the study as key stakeholders in the technology and their views on models' usefulness were collected using a survey.
- Models' assessment focused on clarity of anatomical visualisation, insight into the abnormality, overall perceived usefulness, and comparison to CT scans.
- The study is limited by the small number of participants (n=17 in total), further studies looking at other anomalies may be needed to extrapolate our results to varying classifications of the anomalies.

For peer review only

For peer review only - http://bmjopen.bmj.com/site/about/guidelines.xhtml

Introduction

Three-dimensional (3D) printing offers unique advantages in medicine, with great potential for personalisation in the face of the wide variation of anatomy, morphology and disease across individuals. This technology has seen rapid advancement in its development and applications, with evidence beginning to emerge in support of its effectiveness and clinical value.[1] Benefit has been found in pharmaceutical and interventional research, printing of anatomical models, tissue fabrication, prosthetics and implants.[2] The use of 3D models in teaching, clinical consultations, and pre-operative planning has also been assessed.[1,3,4] The technology has seen a rapid rate of adoption in congenital cardiology, with models aiding understanding of complex anatomical structures and accurately delineating anatomical morphology to make informed management decisions.[5] Areas of positive early experience include pre-operative planning, functional flow models assessing aortic valve dysfunction, device innovation, and teaching.[6,7] Enhance understanding of complex anatomical spatial relationships through patient-personalised 3D models could lead to improving surgical outcomes.[3]

Coronary anatomy has serious implications on cardiac premature morbidity and mortality. Congenital coronary artery anomalies (CAA) present wide variation in morphology, and are prevalent in approximately 1% of the population.[8] They are usually asymptomatic and encountered as an incidental finding during coronary angiography.[9] However, CAAs are the second most common cause of sudden cardiac death in young athletes,[10] e.g. representing the predominant (61%) identifiable cardiac abnormality in a study looking at nontraumatic sudden deaths of young healthy military recruits.[11]

Acquired, non-atherosclerotic coronary artery anomalies can be seen in Kawasaki Disease, a systemic inflammatory condition of medium sized vessels, with a predilection for the coronary arteries.[12] Coronary artery aneurysms are the most common complication of Kawasaki coronary arteritis. Coronary imaging in this population is important for serial follow up, management and

For peer review only - http://bmjopen.bmj.com/site/about/guidelines.xhtml

prognosis. The detection of coronary complications in Kawasaki Disease can be challenging, as usually presents in children, who have small vessel size and increased heart rates.

Coronary computed tomography (CT) angiography is currently the most widely used non-invasive imaging modality for investigating CAA, offering detailed visualisation of the anatomy and coronary arteries with higher sensitivity than invasive coronary angiography.[13,14] A single widely accepted CAA classification scheme is lacking, despite attempts by various groups to sort them by properties including clinical symptoms, anatomical appearance, origin, and risk.[9,10,15,16]

This study thus aims to explore the use of 3D-printed models for viewing and investigating CAAs, evaluating the feasibility of its use both clinically and in an investigative capacity. Two key stakeholders were engaged: a) clinicians, who diagnose CAAs or are involved in management decisions, and b) non-medically qualified cardiovascular researchers, who may benefit from clearer viewing of coronary arteries for research purposes.

Materials and Methods

Case Selection

Two cardiac radiology consultants with >15 years' experience in cardiac CT (NM, MH) selected n=7 cases from our Centre's database of patients with CAA and CT. An additional case with normal coronary anatomy was included ('control case'). The criteria for selecting the cases were:

i) Range of CAA;

ii) Available CT coronary angiogram;

iii) Consent for research use of images.

All scans were displayed on a 512x512 matrix (Table 1).

For peer review only - http://bmjopen.bmj.com/site/about/guidelines.xhtml

Considering the focus of the study on printing feasibility and model evaluation, the study is not considered Clinical Research as defined in the UK Policy Framework for Health and Social Care Research, and HRA and NHS REC approval were not deemed necessary by the local R&D committee.

CT protocol

Cardiac CT scans were performed on a 320-multidetector row CT system (Toshiba Aquilion ONE) or 128-slice cardiac CT (Siemens Medical Solutions, Erlangen, Germany). Patients received 20-60 mL of iodinated contrast (calculated at 20 mg iodine/kg/second for 14-20 seconds), followed by 10-40 mL of normal saline. Scan timing by bolus tracking (Toshiba) and test bolus (Siemens) were performed with region of interest in the ascending aorta.

3D Printing

The anonymised scans were processed with commercial software (Mimics Research 19.0, Materialise NV, Leuven, Belgium) reconstructing the 3D heart model following steps of layer masking, segmentation and region growing.[17] Each scan was assessed by a medical student (ML) with the guidance of a cardiologist with 10 years in cardiac CT (SME) to decide the best way to display the CAA, purposefully removing certain structures to reveal the coronary arteries and abnormality more clearly. The decision to remove parts of the cardiovascular anatomy was made on the basis that the direct line of sight to view and follow the coronary arteries was not obstructed. Models were 1:1 in size. Whilst the accuracy of the reconstruction protocol has been previously demonstrated[17], the resulting 3D reconstruction were also verified visually by the cardiologist against the original CT dataset.

Models were smoothed (3-matic Research 11.0, Materialise) and exported as Stereolithography (STL) files. The STL files were imported into the 3D printing software (PreForm 2.10.3, Formlabs Inc, Somerville, MA, USA) where orientation, scaffolding, and print layout were set before printing the model. The printer (Formlabs Form 2 SLA) was available in-house. Models were all printed in white

For peer review only - http://bmjopen.bmj.com/site/about/guidelines.xhtml

resin. Upon completion of printing, the models were submerged in propanol for 25-30 minutes, then dried in a fume cupboard for an hour. The scaffolding was removed manually. Models were then assessed for quality by a biomedical engineer with 7 years' experience of 3D printing (GB) and checked for correct anatomical representation with respect to the CT scan by the cardiologist.

Models Presentation and Feedback

A questionnaire was devised to collect feedback on the models' usefulness and clarity from two groups of stakeholders (Table 2). Cardiovascular researchers (n=9) had a background in cardiovascular science but had little or no experience of imaging. Clinicians included cardiac surgeons (n=2) and cardiologist (n=6, of which n=3 with extensive experience in cardiac imaging). Cardiologists and radiologists directly involved in patients' selection and model preparation did not take part in the survey.

A presentation was given to the researchers, where all models and their respective CT scans were shown one by one, including basic clinical information for each case (e.g. age, gender, diagnosis, medical history). Screenshots of the CT scans showing coronary anatomy were displayed, followed by introduction of the model which was then passed around and examined. A similar drop-in session was organised for the clinicians to accommodate their less flexible schedules, consisting of the same presentation and feedback questionnaire. The presentation lasted approximately 1 hour. The researcher administering the survey had no prior relationship with participants.

The questionnaire focused on assessing clarity of anatomical visualisation, insight into the abnormality, overall usefulness, and comparison to CT scans. Assessment of model characteristics used Likert-type questions on a 5-point scale (from "Strongly disagree" to "Strongly agree") or a 10-point rating (from 0, lowest score, to 10, highest score). The questionnaire also included a final feedback form summarising overall usefulness and asking for participants' imaging experience (in number of years). One question regarding usefulness to future practice/applications was modified slightly to suit either research or clinical practice (i.e. usefulness "as part of your clinical

For peer review only - http://bmjopen.bmj.com/site/about/guidelines.xhtml

practice"/"as part of your research"). Stakeholders were asked to fill in a feedback form following presentation of each case and a final feedback form.

Data analysis

Analysis of questionnaire responses was carried out in Stata (v.13, StataCorp LLC, College Station, TX, USA). Responses are presented as counts or mean±SD as appropriate. Unpaired comparisons between the two study groups (clinicians vs. researchers) were carried out using Wilcoxon rank sum test. Open-ended feedback was qualitatively analysed for dominant themes.

Patient and public involvement

The study involved key stakeholders in the technology (i.e. clinicians and researchers), according to a model of social construction of technology. In this instance, patients were not involved as beyond the specific focus of the study, despite being key stakeholders in the technology.

Results

The CT scans and models of each case are shown (Figure 1). CT scans were of adequate quality for segmentation. All models were reconstructed and printed successfully. Details of each printed model are reported (Table 3). All questionnaires were completed successfully apart from one incomplete in the researchers group.

Coronary artery anatomy was interpreted clearly by both groups and coronary artery anomalies were identified clearly on the models by clinicians (8.9±0.8/10) and researchers (7.4±0.3/10). The abnormality was only missed in 2% of cases overall. Clinicians tended to rate the models slightly higher than researchers on average in terms of clarity of anatomy (8.9±0.8 vs. 7.5±0.4, p=0.02), abnormality (9.0±0.7 vs. 7.5±0.5, p=0.03) and usefulness (9.1±0.7 vs. 7.7±0.5, p=0.03).

For peer review only - http://bmjopen.bmj.com/site/about/guidelines.xhtml

Looking at both groups combined and accounting for any incomplete answer, in 98% of cases (126/130 cases), models enhanced the awareness of coronary anatomy and abnormality. Clinicians found the models more useful over viewing the CT scans alone (4.5±0.3/5), as did researchers (4.2±0.3 /5). Almost all participants (16/17) stated that the models would be useful in any future work involving coronary artery anomalies. The dominant themes emerging from clinicians' and researchers' comments are reported in Table 4. Evaluation of model usefulness is reported in detail in Table 5.

Clinicians

All clinicians had at least moderate expertise to view and interpret scans, with 5 of them having advanced imaging expertise and working with scans daily. Generally, clinicians found the coronary arteries easy to follow and reported to easily identify the anatomy and abnormality. In 89% of all cases, they agreed that the 3D model was more effective for their understanding of the abnormality than just looking at the CT scan, with 74% stating they 'strongly agreed'. Clinicians rated the models for case 6 (Transposition of Great Arteries with abnormal circumflex artery) and case 8 (Anomalous Left Coronary Artery from Pulmonary Artery) the clearest and most effective. All clinicians agreed that models would be useful in their future work with coronary artery anomalies and suggested use for:

- Junior and senior radiologists, to demonstrate the diagnostic CT anatomy to clinicians
- Explaining conditions to patients
- Surgeons, during pre-operative planning

Researchers

In most cases (7/9) researchers had only basic imaging expertise or less, with 5 of them having no imaging expertise. In general, researchers could interpret the model well, but their scores of clarity of anatomy and clarity of abnormality were slightly less enthusiastic than clinicians' scores. In 97% of

For peer review only - http://bmjopen.bmj.com/site/about/guidelines.xhtml

cases, researchers agreed that viewing the 3D model was more effective for their understanding than just looking at the CT scan, with 17% ‘strongly agreeing’ it was more effective. Each researcher reported that in an average of 69% of cases the models enhanced their anatomical awareness, and that the course of coronary arteries was easy to follow. Researchers gave the highest ratings to case 6 (TGA), case 7 (Kawasaki’s Disease coronary aneurysm, Figure 2) and case 8 (ALCAPA, Figure 3). The open-ended feedback revealed that researchers found the models a bit more difficult to interpret, but 8/9 agreed they would be useful in any future work studying coronary artery anomalies.

Researchers interestingly remarked on the potential for clinical applications of the models and particularly mentioned:

- Surgeons in making pre-surgical decisions;
- Clinicians to make management decisions;
- Helping patients and families understand disease.

Discussion

Considering the complexity of coronary artery anomalies, patient-specific models can provide enhanced visuospatial appreciation of the defect in relation to other anatomical structures and increase the user’s understanding of the anatomy, as shown in this study. Enhanced anatomical appreciation has been proven to lead to better pre-operative planning and management decisions within a clinical setting[18]. This appreciation of relational structures is particularly vital in the clinical picture of CAAs, where evaluation of the nature, location and course to determine degree of abnormality of the CAA and risk of harm to the patient can significantly alter management decisions.[9,10,19]

3D printing has been used to reconstruct and print the coronary arterial tree[20] and it has been demonstrated that 3D printing models were effective in visualising the coronary arteries.[5] At

For peer review only - http://bmjopen.bmj.com/site/about/guidelines.xhtml

present, only two case reports present 3D printed models for the evaluation of coronary fistulae.[21,22] In these cases, the 3D-printed model anecdotally added value in management decisions. However, no studies have been carried out to evaluate clinical use of 3D printed models for coronary arteries and CAAs.

Currently, CAAs are investigated primarily by CT angiography, which has shown to have an excellent detection rate and is fast becoming the imaging modality of choice.[13,14] Although the CT scans may adequately delineate coronary anatomy, our study showed that the complementary use of 3D printed models was useful for viewing coronary anatomy and anomalies by not only researchers but also clinicians with imaging and cardiovascular background, and was considered effective for increasing their understanding of the abnormality over simply viewing the CT scans. A randomised controlled trial by Li et al. recently drew similar conclusions in comparing CT scans vs. 3D modalities for teaching purposes. In this case, 2D CT images, monochromatic 3D virtual models and monochromatic 3D printed models were used for teaching cervical and thoracic spinal anatomy and fractures. A 10-mark post-teaching examination showed that students with teaching using 3D-virtual and 3D-printed models scored higher than students in the CT group, demonstrating efficacy of 3D learning methods over 2D.[23]

For physicians, the use of a patient-specific model is preferable to looking at CT and MRI scans as they are viewed on a 2D flat screen.[24] This indicates that 3D printed models may bring an added aspect to multidisciplinary meeting discussions, perhaps due to the ability to hold and rotate the model from different angles to appreciate dimensions and spatial relationships.

In our study, reconstruction and printing of the 3D heart models required a significant amount of resources and time. The average time for reconstruction and printing was 27±1.7 hours. This was practical in a research environment with less resource and time constraints; however, if models were to be used in the clinical setting then this may require a dedicated team operating the 3D printers for such a process to be feasible.

For peer review only - http://bmjopen.bmj.com/site/about/guidelines.xhtml

Both clinicians and researchers found the models clear and effective in displaying coronary anatomy and the anomalies, however clinicians rated the models higher on average. This difference may be explained by the intrinsic differences between the two groups, where clinicians have a stronger background in viewing imaging and anatomical understanding. This is reflected in their self-declared measure of imaging expertise, whereas 7 out of 9 researchers had only basic imaging expertise. As a result, clinicians likely have better understanding of coronary artery course and relational structures, and may be able to better appreciate the anatomy displayed by the model. Conversely, the models were still useful for researchers who were unable to fully understand the pathology from CT scans but identified it on the models. These findings suggest that the models are more effectively interpreted by users with a stronger ability to understand relevant anatomy, but still hold purposeful value for individuals with less expertise.

Both clinicians and researchers found the models of Transposition of Great Arteries (TGA) with abnormal circumflex artery and of Anomalous Left Coronary Artery from Pulmonary Artery (ALCAPA) the most effective and clearest to understand. Interestingly, comments by three clinicians suggested that the pathology for the ALCAPA case was difficult to see on CT scans, whereas the anatomy was clearly delineated on the 3D printed models. Consideration of the complexity of the anatomical differences associated with the disease –TGA and ALCAPA being congenital coronary variations with abnormal coronary origin and course– suggest that the two conditions are more difficult to understand and follow than the pathologies of other cases which scored slightly lower. 3D printed models may potentially confer the most benefit in complex pathologies; this is consistent with the results of a study where anatomical teaching for the three most complicated regions of the body was delivered effectively with 3D modelling techniques.[25]

Segmentation of the models with removal of certain structures was to some extent an operator-dependent procedure. It was, in general, met with good response. 80% of clinicians and 62% researchers reported that it facilitated viewing the anatomy and the abnormality, whilst only 5% of

For peer review only - http://bmjopen.bmj.com/site/about/guidelines.xhtml

clinicians and researchers stated that it made it harder to appreciate the anatomy and abnormality. Two clinicians questioned whether removing parts of the heart was the best approach, as in real life "we don't have parts removed". Although no formal review has been done of effectiveness of removing structures during segmentation process, it is a technique that has been used previously.[21,22] One clinician suggested using a more flexible material that enables structures to be bent to view the underlying coronary arteries, and there may be scope in further investigations to trial more flexible materials such as TangoPlus FullCure, which has been used for preoperative surgical training.[25,26] Another option is the use of silicone models. Flexible models can be created with an injection molding technique using infusion of gel materials such as silicone and urethane into the mold.[27] A more versatile material resulting in bendable structures may allow for preservation of cardiac anatomy and structures, and manual manipulation to view the coronary arteries and anomalies.

One further suggestion by both clinicians and researchers was the use of colour to distinguish coronary arteries from other structures. The colour of certain structures can be "painted" on the digital file before printing, as demonstrated by Mcmenamin et al. who reconstructed prosections of the hand and wrist and highlighted various anatomical features using the software package 3D-Coat (Kompaniya Pilgway Studio, Ukraine). This was shown to create realistic 3D replicas in which "even small nerves and vessels could be readily distinguished".[28] The in-house printer used for our study did not support printing with multiple colours, and further studies can be conducted with higher-end printers to evaluate the use of colour in looking at coronary arteries anomalies.

The cases for this study were selected from a hospital database, thus the overall representation of CAAs in this study may be skewed towards more symptomatically severe cases requiring medical attention. Our results may therefore not be generalisable to CAAs of all severities. However, uncomplicated cases of CAA not necessitating treatment would likely not benefit significantly from a 3D printed model.

For peer review only - http://bmjopen.bmj.com/site/about/guidelines.xhtml

Our study was limited by the relatively small number of cases, and although a spectrum of coronary artery anomalies was successfully produced, others were not assessed e.g. atresia, congenital absence and duplication. An appreciation of the significant haemodynamic and structural differences of the different anomalies suggests that further studies looking at other anomalies may need to be done to extrapolate our results to varying classifications of the anomalies.[16] Furthermore, it would be interesting to explore differences between sub-groups, particularly between clinicians with and without imaging expertise, whereas in this study this was limited by the small sample size of participants and the focus was comparing participants with and without medical training.

Wider future use of 3D printing in coronary artery anomalies may require a more standardised protocol in production and quality assessment. Independent review of the models and accuracy to scan was done in this study, showing accurate reconstruction of the models and coronary arteries. A formal method of assessment such as Bland-Altman analysis –quantifying agreement between the dimensions of CT scans and 3D models– has been used to confirm accuracy of the 3D printed models.[29,30] These can be used in the future to verify consistency.

Conclusion

Where small discrepancies in coronary anatomy can be significant in determining prognosis and presentation, it is important that the exact anatomy should be fully understood before management decisions can be appropriately made. 3D-printed heart models can be feasibly used to recreate coronary artery anatomy and enhance understanding of the abnormality. The models could complement CT scans to display anatomy and abnormalities, and in more anatomically complex cases display structural relationships not entirely clear on the scans for users with moderate or no expertise in viewing imaging data. Future studies toward the use of 3D printing in coronary artery anomalies should investigate the best purpose of use of 3D-printed models within the spectrum of coronary artery anomalies, in terms of severity and classification. There may be scope for use in pre-

For peer review only - http://bmjopen.bmj.com/site/about/guidelines.xhtml

operative planning and decision-making, as well as teaching and clinical consultations. Cost-benefit should be determined before its role can be established alongside conventional imaging alternatives for investigating coronary artery anomalies such as CT angiography.

Conflict of interest

The authors declare there was no conflict of interest upon conduction of study.

Acknowledgements and Funding

The work is supported by a David Telling Charitable Trust equipment grant and by the British Heart Foundation. The authors also acknowledge support from the Bristol National Institute for Health Research (NIHR) Biomedical Research Centre. The views expressed in this publication are those of the authors and not necessarily those of the NHS, the National Institute for Health Research or the Department of Health.

Author Contributions

ML created the models and carried out the survey, with RB, as well as analysing survey results. SME, MH, NM, EGM, CBD and MC aided with selection of cases, clinical insight and model checking. GB conceived the study and oversaw the creation of models and the analysis. ML drafted the manuscript with GB and all authors critically contributed to revising and finalising the manuscript.

Data Sharing Statement

All data pertinent to the study are included in the manuscript, no additional unpublished data.

For peer review only - http://bmjopen.bmj.com/site/about/guidelines.xhtml

For peer review only

References

1. Dodziuk, H., 2016. Applications of 3D printing in healthcare. Kardiochir Torakochirurgia Pol 13, 283–293. https://doi.org/10.5114/kitp.2016.62625
2. Ventola, C.L., 2014. Medical Applications for 3D Printing: Current and Projected Uses. P T 39, 704–711.
3. Bhatla, P., Tretter, J.T., Ludomirsky, A., Argilla, M., Latson, L.A., Chakravarti, S., Barker, P.C., Yoo, S.-J., McElhinney, D.B., Wake, N., Mosca, R.S., 2017. Utility and Scope of Rapid Prototyping in Patients with Complex Muscular Ventricular Septal Defects or Double-Outlet Right Ventricle: Does it Alter Management Decisions? Pediatr Cardiol 38, 103–114. https://doi.org/10.1007/s00246-016-1489-1
4. Loke, Y.-H., Harahsheh, A.S., Krieger, A., Olivieri, L.J., 2017. Usage of 3D models of tetralogy of Fallot for medical education: impact on learning congenital heart disease. BMC Medical Education 17, 54. https://doi.org/10.1186/s12909-017-0889-0
5. Javan, R., Herrin, D., Tangestanipoor, A., 2016. Understanding Spatially Complex Segmental and Branch Anatomy Using 3D Printing: Liver, Lung, Prostate, Coronary Arteries, and Circle of Willis. Acad Radiol 23, 1183–1189. https://doi.org/10.1016/j.acra.2016.04.010
6. Bartel, T., Rivard, A., Jimenez, A., Mestres, C.A., Müller, S., 2017. Medical three-dimensional printing opens up new opportunities in cardiology and cardiac surgery. Eur. Heart J. https://doi.org/10.1093/eurheartj/ehx016
7. Vukicevic, M., Mosadegh, B., Min, J.K., Little, S.H., 2017. Cardiac 3D Printing and its Future Directions. JACC Cardiovasc Imaging 10, 171–184. https://doi.org/10.1016/j.jcmg.2016.12.001
8. Yildiz, A., Okcun, B., Peker, T., Arslan, C., Olcay, A., Bulent Vatan, M., 2010. Prevalence of Coronary Artery Anomalies in 12,457 Adult Patients Who Underwent Coronary Angiography. Clin Cardiol 33, E60–E64. https://doi.org/10.1002/clc.20588
9. Ali, M., Hanley, A., McFadden, E.P., Vaughan, C.J., 2011. Coronary artery anomalies: a practical approach to diagnosis and management. Heart Asia 3, 8–12. https://doi.org/10.1136/ha.2010.003244
10. Villa, A.D., Sammut, E., Nair, A., Rajani, R., Bonamini, R., Chiribiri, A., 2016. Coronary artery anomalies overview: The normal and the abnormal. World J Radiol 8, 537–555. https://doi.org/10.4329/wjr.v8.i6.537
11. Eckart, R.E., Scoville, S.L., Campbell, C.L., Shry, E.A., Stajduhar, K.C., Potter, R.N., Pearse, L.A., Virmani, R., 2004. Sudden death in young adults: a 25-year review of autopsies in military recruits. Ann. Intern. Med. 141, 829–834.
12. Kawasaki, T., Kosaki, F., Okawa, S., Shigematsu, I., and Yanagawa, H. A new infantile acute febrile mucocutaneous lymph node syndrome (MLNS) prevailing in Japan. Pediatrics. 1974; 54: 271–276.
13. Ghadri, J.R., Kazakauskaite, E., Braunschweig, S., Burger, I.A., Frank, M., Fiechter, M., Gebhard, C., Fuchs, T.A., Templin, C., Gaemperli, O., Lüscher, T.F., Schmied, C., Kaufmann, P.A., 2014. Congenital coronary anomalies detected by coronary computed tomography compared to invasive coronary angiography. BMC Cardiovascular Disorders 14, 81. https://doi.org/10.1186/1471-2261-14-81
14. Budoff, M.J., Ahmed, V., Gul, K.M., Mao, S.S., Gopal, A., 2006. Coronary anomalies by cardiac computed tomographic angiography. Clin Cardiol 29, 489–493. https://doi.org/10.1002/clc.4960291104
15. Dodge-Khatami, A., Mavroudis, C., Backer, C.L., 2000. Congenital Heart Surgery Nomenclature and Database Project: anomalies of the coronary arteries. Ann. Thorac. Surg. 69, S270-297.
16. Shriki, J.E., Shinbane, J.S., Rashid, M.A., Hindoyan, A., Withey, J.G., DeFrance, A., Cunningham, M., Oliveira, G.R., Warren, B.H., Wilcox, A., 2012. Identifying, characterizing,

For peer review only - http://bmjopen.bmj.com/site/about/guidelines.xhtml

and classifying congenital anomalies of the coronary arteries. Radiographics 32, 453–468. https://doi.org/10.1148/rg.322115097

17. Schievano, S., Migliavacca, F., Coats, L., Khambadkone, S., Carminati, M., Wilson, N., Deanfield, J.E., Bonhoeffer, P., Taylor, A.M., 2007. Percutaneous pulmonary valve implantation based on rapid prototyping of right ventricular outflow tract and pulmonary trunk from MR data. Radiology 242, 490–497. https://doi.org/10.1148/radiol.2422051994
18. Smith, M.L., McGuinness, J., O'Reilly, M.K., Nolke, L., Murray, J.G., Jones, J.F.X., 2017. The role of 3D printing in preoperative planning for heart transplantation in complex congenital heart disease. Ir J Med Sci. https://doi.org/10.1007/s11845-017-1564-5
19. Reul, R.M., Cooley, D.A., Hallman, G.L., Reul, G.J., 2002. Surgical Treatment of Coronary Artery Anomalies. Tex Heart Inst J 29, 299–307.
20. Hinton, T.J., Jallerat, Q., Palchesko, R.N., Park, J.H., Grodzicki, M.S., Shue, H.-J., Ramadan, M.H., Hudson, A.R., Feinberg, A.W., 2015. Three-dimensional printing of complex biological structures by freeform reversible embedding of suspended hydrogels. Science Advances 1, e1500758. https://doi.org/10.1126/sciadv.1500758
21. Velasco Forte, M.N., Byrne, N., Valverde Perez, I., Bell, A., Gómez-Ciriza, G., Krasemann, T., Sievert, H., Simpson, J., Pushparajah, K., Razavi, R., Qureshi, S., Hussain, T., 2017. 3D printed models in patients with coronary artery fistulae: anatomical assessment and interventional planning. EuroIntervention 13, e1080–e1083. https://doi.org/10.4244/EIJ-D-16-00897
22. Aroney, N., Lau, K., Daniele, L., Burstow, D., Walters, D., 2017. Three-dimensional printing: to guide management of a right coronary artery to left ventricular fistula. Eur Heart J Cardiovasc Imaging. https://doi.org/10.1093/ehjci/jex317
23. Li, Zhenzhu, Li, Zefu, Xu, R., Li, M., Li, J., Liu, Y., Sui, D., Zhang, W., Chen, Z., 2015. Three-dimensional printing models improve understanding of spinal fracture--A randomized controlled study in China. Sci Rep 5, 11570. https://doi.org/10.1038/srep11570
24. Gross, B.C., Erkal, J.L., Lockwood, S.Y., Chen, C., Spence, D.M., 2014. Evaluation of 3D printing and its potential impact on biotechnology and the chemical sciences. Anal. Chem. 86, 3240–3253. https://doi.org/10.1021/ac403397r
25. Pujol, S., Baldwin, M., Nassiri, J., Kikinis, R., Shaffer, K., 2016. Using 3D Modeling Techniques to Enhance Teaching of Difficult Anatomical Concepts. Acad Radiol 23, 507–516. https://doi.org/10.1016/j.acra.2015.12.012
26. Yoo, S.-J., Spray, T., Austin, E.H., Yun, T.-J., van Arsdell, G.S., 2017. Hands-on surgical training of congenital heart surgery using 3-dimensional print models. J. Thorac. Cardiovasc. Surg. 153, 1530–1540. https://doi.org/10.1016/j.jtcvs.2016.12.054
27. Shiraishi, I., Yamagishi, M., Hamaoka, K., Fukuzawa, M., Yagihara, T., 2010. Simulative operation on congenital heart disease using rubber-like urethane stereolithographic biomodels based on 3D datasets of multislice computed tomography. Eur J Cardiothorac Surg 37, 302–306. https://doi.org/10.1016/j.ejcts.2009.07.046
28. McMenamin, P.G., Quayle, M.R., McHenry, C.R., Adams, J.W., 2014. The production of anatomical teaching resources using three-dimensional (3D) printing technology. Anat Sci Educ 7, 479–486. https://doi.org/10.1002/ase.1475
29. Valverde, I., Gomez, G., Suarez-Mejias, C., Hosseinpour, A.-R., Hazekamp, M., Roest, A., Vazquez-Jimenez, J.F., El-Rassi, I., Uribe, S., Gomez-Cia, T., 2015. 3D printed cardiovascular models for surgical planning in complex congenital heart diseases. J Cardiovasc Magn Reson 17. https://doi.org/10.1186/1532-429X-17-S1-P196
30. Olejník, P., Nosal, M., Havran, T., Furdova, A., Cizmar, M., Slabej, M., Thurzo, A., Vitovic, P., Klvac, M., Acel, T., Masura, J., 2017. Utilisation of three-dimensional printed heart models for operative planning of complex congenital heart defects. Kardiol Pol 75, 495–501. https://doi.org/10.5603/KP.a2017.0033

For peer review only - http://bmjopen.bmj.com/site/about/guidelines.xhtml

Table 1: Acquisition and clinical information of patients and their scans for each case.

CASES	ACQUISITION INFORMATION			CLINICAL INFORMATION		
	Scanner	Slice Thickness (mm)	Detectors	Age (y)	Gender	Pathology
**Case 1**	Toshiba Aquilion ONE	0.50	320	39	M	Normal coronary anatomy
**Case 2**	Toshiba Aquilion ONE	0.50	320	13	F	Multiple anomalous coronary arteries
**Case 3**	Toshiba Aquilion ONE	0.50	320	10	F	Coronary fistula
**Case 4**	Toshiba Aquilion ONE	0.50	320	36	M	Myocardial bridging of coronary artery
**Case 5**	SOMATOM Definition AS+	0.75	128	48	M	Tetralogy of Fallot; LCX and LAD come off separately from aorta
**Case 6**	Toshiba Aquilion ONE	0.50	320	18	M	Transposition of Great Arteries; Abnormal circumflex artery
**Case 7**	SOMATOM Definition AS+	0.75	128	52	F	Kawasaki's disease; Left main stem coronary artery aneurysm
**Case 8**	Toshiba Aquilion ONE	0.50	320	56	F	Anomalous left coronary artery from pulmonary artery (ALCAPA)

For peer review only

For peer review only - http://bmjopen.bmj.com/site/about/guidelines.xhtml

Table 2: Purposes for inclusion of various specialties within clinician group.

Party	Purpose for Inclusion in Feedback	How would 3D Modelling be Useful to this Party?
Researcher	Strong cardiovascular background but likely limited experience with cardiac imaging and congenital heart disease	Appreciation of anatomy across different congenital coronary artery diseases; potential use of models in future research
Cardiac surgeon	Experienced with cardiac anatomy, pathology and imaging; specific focus on potential use of models for pre-operative assessment	Understanding specific visuospatial anatomy abnormality; pre-operative planning and practice
Radiologist/ imaging expert	Extremely experienced with imaging; assessing whether 3D models improve/complement appreciation from CT alone	Visuospatial appreciation of abnormality in addition to what they see on scans
Cardiologist	Experienced with cardiac anatomy, pathology and imaging; assessing whether models can improve/complement understanding	Use for complex cases with difficult coronary anatomy; support management decisions based on measurable dimensions

For peer review only - http://bmjopen.bmj.com/site/about/guidelines.xhtml

Table 3: Details of each printed model.

Case	Reconstruction time (hrs)	Resin Volume (mL)	Layers (n)	Print Duration
Case 1	4.5	139.2	3202	22 hrs 51 min
Case 2	6	88.36	2498	14 hrs 24 min
Case 3	5	81.71	2282	12 hrs 43 min
Case 4	9	130.6	2626	29 hrs 30 min
Case 5	7	214.3	3225	33 hrs 05 min
Case 6	6.5	107.3	2683	17 hrs 45 min
Case 7	4.5	76.70	2479	13 hrs 45 min
Case 8	4	154.0	3334	25 hrs 01 min

For peer review only

Table 4: Dominant themes emerging from comments received by clinicians and researchers

Clinicians' Comments	n	Researchers' Comments	n
Enhanced visuospatial awareness using the model	5	Model was easier to understand after some guidance	4
It was useful to remove parts of structures to follow the coronary arteries	3	Removal of certain parts useful to give focus on the part of interest	2
Models would be effective for training of postgraduates and medical students	3	Use of colouring to delineate coronary arteries and abnormalities would make model more effective	2
ALCAPA (Case 8) was difficult to identify on the CT scans, but the anatomy was clear on the model	3	Understanding of anatomy improved as more models looked at	1
Use of colouring to delineate coronary arteries and abnormalities would make model more effective	2		

For peer review only

Case	Diagnosis	Clarity of Anatomy Average (out of 10)			Clarity of Abnormality Average (out of 10)			Usefulness Average (out of 10)			Effectiveness over CT Scan (from 1 = strongly disagree to 5 = strongly agree)		
		**Clinicians**	**Researchers**	**p value**	**Clinicians**	**Researchers**	**p value**	**Clinicians**	**Researchers**	**p value**	**Clinicians**	**Researchers**	**p value**
**2**	Multiple anomalous coronaries	8.9±1.5	6.9±1.5	0.021*	9.4±1.4	6.9±1.5	0.006*	9.2±1.4	6.7±1.6	0.009*	4.6±0.7	3.9±0.6	0.036*
**3**	Coronary fistula	7.5±3.0	7.0±1.1	0.520	7.9±2.7	6.5±1.8	0.108	8.0±2.8	7.1±1.1	0.142	4.1±0.8	4.0±0	0.607
**4**	Bridging	8.2±2.8	7.4±0.8	0.072	8.1±2.7	7.4±0.7	0.068	8.4±2.7	7.3±0.9	0.033*	4.4±1.1	4.3±0.5	0.365
**5**	Separate LCX and LAD from aorta	9.2±1.4	7.2±1.2	0.010*	9.2±1.4	7.0±1.8	0.010*	9.1±1.4	7.0±1.6	0.013	4.2±0.7	3.8±0.7	0.158
**6**	TGA, abnormal circumflex	9.5±0.7	7.5±0.9	0.002*	9.4±0.9	7.5±0.8	0.003*	9.5±0.8	7.6±0.8	0.003*	4.8±0.5	4.4±0.5	0.221
**7**	Kawasaki's aneurysm	9.2±1.4	8.1±1.1	0.041*	9.4±1.4	8.2±1.0	0.027*	9.2±1.4	8.4±0.9	0.055	4.6±0.7	4.4±0.5	0.346
**8**	ALCAPA	10.0±0	7.8±0.7	<0.001 *	9.9±0.3	7.8±1.0	<0.001*	9.9±0.3	8.1±0.6	<0.001*	5.0±0	4.3±0.5	0.005*

Table 5: Evaluation of model usefulness. LCX = ; LAD = ; TGA = transposition of the great arteries; ALCAPA = anomalous left coronary artery from pulmonary artery

For peer review only - http://bmjopen.bmj.com/site/about/guidelines.xhtml

Figure Legends

Figure 1: CT Scans and models of each case, with the blue arrows pointing to the abnormality on the CT. Case 1 (1st row left 1.1-1.3): Normal coronary anatomy; axial view progressing inferiorly (1.1,1.2). Case 2 (1st row right 2.1-2.3): Multiple anomalous coronary arteries; axial view progressing inferiorly (2.1,2.2). Case 3 (2nd row left 3.1-3.3): Coronary fistula; Sagittal view progressing right (3.1,3.2). Case 4 (2nd row right 4.1-4.3): Myocardial bridging; Axial view progressing inferiorly (4.1,4.2). Case 5 (3rd row left 5.1-5.3): Separate LCX and LAD from aorta, tetralogy of Fallot; Coronal view (5.1) and Axial view (5.2). Case 6 (3rd row right 6.1-6.3): Transposition of Great Arteries (TGA) with abnormal circumflex artery; Axial view progressing inferiorly (6.1,6.2). Case 7 (4th row left 7.1-7.3): Kawasaki ' s disease with left main stem coronary artery aneurysm; Coronal view (7.1), Axial view (7.2). Case 8 (4th row right 8.1-8.3): Anomalous left coronary artery from pulmonary artery (ALCAPA); Axial view progressing inferiorly (8.1,8.2).

Figure 2: Detail of Kawasaki model for appreciation of coronary aneurysm (indicated by yellow arrow), two different views.

Figure 3: Detail of ALCAPA model for appreciation of coronary ostium in the pulmonary artery (A) and course of the LCA (B), as indicated by the yellow arrows.

For peer review only - http://bmjopen.bmj.com/site/about/guidelines.xhtml

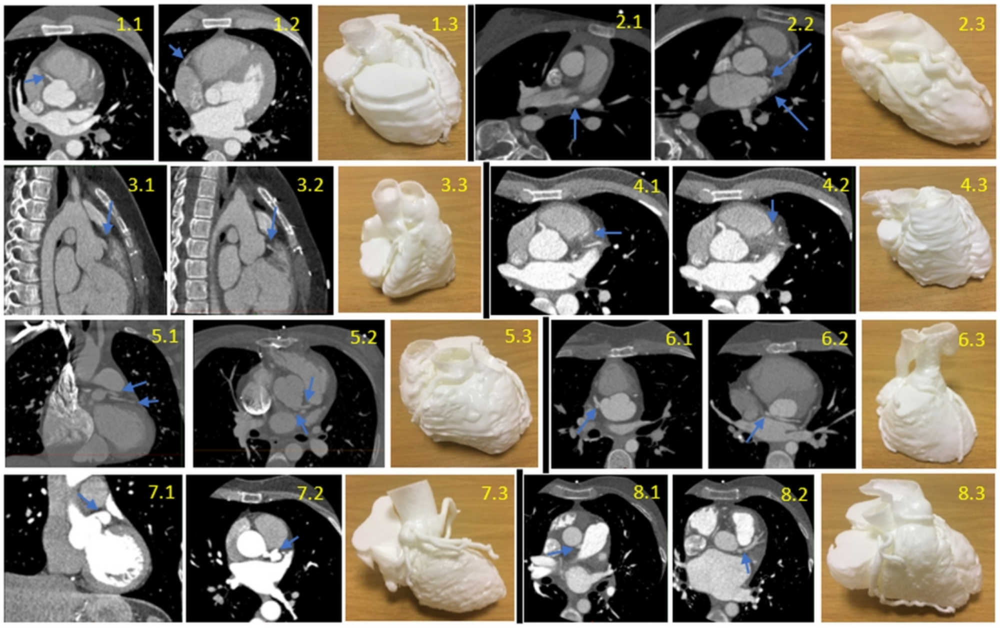

Figure 1

143x90mm (300 x 300 DPI)

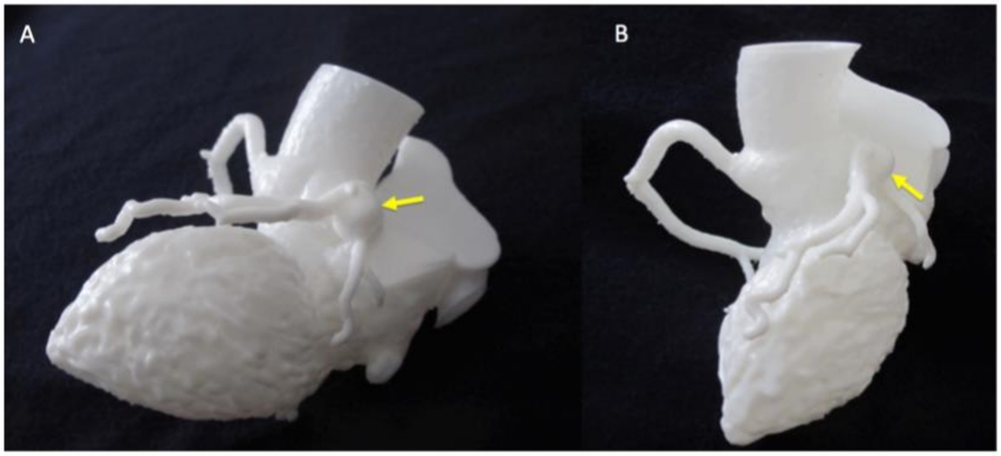

Figure 2

197x90mm (300 x 300 DPI)

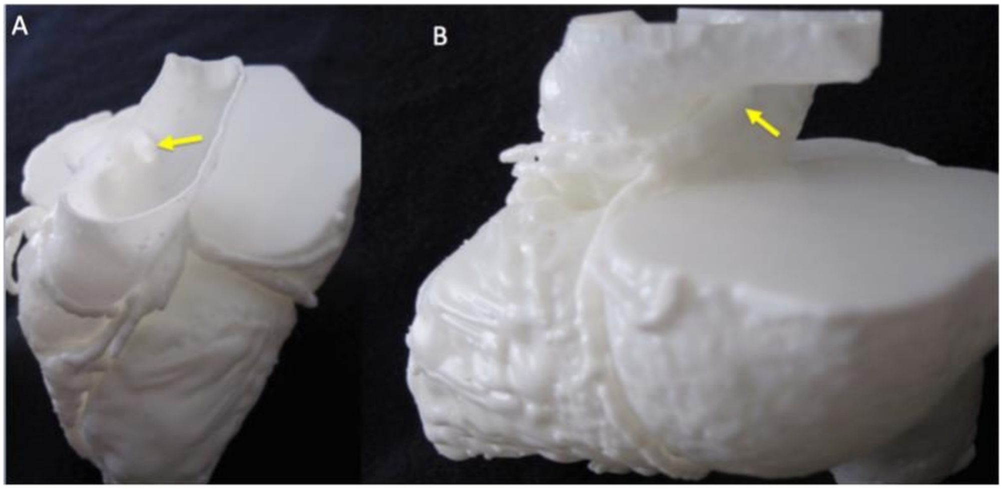

Figure 3

184x90mm (300 x 300 DPI)

Reporting checklist for qualitative study.

Based on the SRQR guidelines.

Instructions to authors

Complete this checklist by entering the page numbers from your manuscript where readers will find each of the items listed below.

Your article may not currently address all the items on the checklist. Please modify your text to include the missing information. If you are certain that an item does not apply, please write "n/a" and provide a short explanation.

Upload your completed checklist as an extra file when you submit to a journal.

In your methods section, say that you used the SRQR reporting guidelines, and cite them as:

O'Brien BC, Harris IB, Beckman TJ, Reed DA, Cook DA. Standards for reporting qualitative research: a synthesis of recommendations. Acad Med. 2014;89(9):1245-1251.

		Reporting Item	Page Number
	#1	Concise description of the nature and topic of the study identifying the study as qualitative or indicating the approach (e.g. ethnography, grounded theory) or data collection methods (e.g. interview, focus group) is recommended	1
	#2	Summary of the key elements of the study using the abstract format of the intended publication; typically includes background, purpose, methods, results and conclusions	2
Problem formulation	#3	Description and signifcance of the problem / phenomenon studied: review of relevant theory and empirical work; problem statement	5
Purpose or research question	#4	Purpose of the study and specific objectives or questions	6
Qualitative approach and research paradigm	#5	Qualitative approach (e.g. ethnography, grounded theory, case study, phenomenolgy, narrative research) and	8

For peer review only

		guiding theory if appropriate; identifying the research paradigm (e.g. postpositivist, constructivist / interpretivist) is also recommended; rationale. The rationale should briefly discuss the justification for choosing that theory, approach, method or technique rather than other options available; the assumptions and limitations implicit in those choices and how those choices influence study conclusions and transferability. As appropriate the rationale for several items might be discussed together.	
Researcher characteristics and reflexivity	#6	Researchers' characteristics that may influence the research, including personal attributes, qualifications / experience, relationship with participants, assumptions and / or presuppositions; potential or actual interaction between researchers' characteristics and the research questions, approach, methods, results and / or transferability	8
Context	#7	Setting / site and salient contextual factors; rationale	8
Sampling strategy	#8	How and why research participants, documents, or events were selected; criteria for deciding when no further sampling was necessary (e.g. sampling saturation); rationale	Table 2
Ethical issues pertaining to human subjects	#9	Documentation of approval by an appropriate ethics review board and participant consent, or explanation for lack thereof; other confidentiality and data security issues	6
Data collection methods	#10	Types of data collected; details of data collection procedures including (as appropriate) start and stop dates of data collection and analysis, iterative process, triangulation of sources / methods, and modification of procedures in response to evolving study findings; rationale	8
Data collection instruments and technologies	#11	Description of instruments (e.g. interview guides, questionnaires) and devices (e.g. audio recorders) used for data collection; if / how the instruments(s) changed over the course of the study	8
Units of study	#12	Number and relevant characteristics of participants, documents, or events included in the study; level of	8

For peer review only

		participation (could be reported in results)	
Data processing	#13	Methods for processing data prior to and during analysis, including transcription, data entry, data management and security, verification of data integrity, data coding, and anonymisation / deidentification of excerpts	8
Data analysis	#14	Process by which inferences, themes, etc. were identified and developed, including the researchers involved in data analysis; usually references a specific paradigm or approach; rationale	9
Techniques to enhance trustworthiness	#15	Techniques to enhance trustworthiness and credibility of data analysis (e.g. member checking, audit trail, triangulation); rationale	n/a
Syntheses and interpretation	#16	Main findings (e.g. interpretations, inferences, and themes); might include development of a theory or model, or integration with prior research or theory	9-10
Links to empirical data	#17	Evidence (e.g. quotes, field notes, text excerpts, photographs) to substantiate analytic findings	Table 4
Intergration with prior work, implications, transferability and contribution(s) to the field	#18	Short summary of main findings; explanation of how findings and conclusions connect to, support, elaborate on, or challenge conclusions of earlier scholarship; discussion of scope of application / generalizability; identification of unique contributions(s) to scholarship in a discipline or field	11-13
Limitations	#19	Trustworthiness and limitations of findings	14
Conflicts of interest	#20	Potential sources of influence of perceived influence on study conduct and conclusions; how these were managed	15
Funding	#21	Sources of funding and other support; role of funders in data collection, interpretation and reporting	15

The SRQR checklist is distributed with permission of Wolters Kluwer © 2014 by the Association of American Medical Colleges. This checklist was completed on 03. June 2018 using http://www.goodreports.org/, a tool made by the EQUATOR Network in collaboration with Penelope.ai

For peer review only

BMJ Open

Evaluating 3D printed models of coronary anomalies: a survey amongst clinicians and researchers

Journal:	*BMJ Open*
Manuscript ID	bmjopen-2018-025227.R1
Article Type:	Research
Date Submitted by the Author:	30-Nov-2018

Complete List of Authors:	Lee, Matthew ; University of Bristol School of Clinical Science<br>Moharem-Elgamal, Sarah; University of Bristol, Bristol Heart Institute<br>Beckingham, Rylan; University of Bristol<br>Hamilton, Mark; University Hospitals Bristol NHS Foundation Trust<br>Manghat, Nathan; University Hospitals Bristol NHS Foundation Trust<br>Milano, Elena; Universita di Verona<br>Bucciarelli Ducci, Chiara; University of Bristol<br>Caputo, Massimo; University of Bristol, Bristol Heart Institute<br>Biglino, Giovanni; University of Bristol School of Clinical Science, Bristol Heart Institute

**Primary Subject Heading**:	Cardiovascular medicine
Secondary Subject Heading:	Communication, Radiology and imaging
Keywords:	Coronary artery anomalies, 3D printing, Computed tomography < RADIOLOGY & IMAGING, coronary arteries

SCHOLARONE™
Manuscripts

Evaluating 3D Printed Models of Coronary Anomalies:
A survey Amongst Clinicians and Researchers

Matthew Lee[1], Sarah Moharem-Elgamal[2,3], Rylan Beckingham[3], Mark Hamilton[4], Nathan Manghat[4], Elena Giulia Milano[3,5], Chiara Bucciarelli-Ducci[3], Massimo Caputo[3], and Giovanni Biglino[3,6]

[1]Bristol Medical School, University of Bristol, Bristol, UK ; [2]National Heart Institute, Giza, Egypt; [3]Bristol Heart Institute, University of Bristol, Bristol, UK; [4]Radiology Department, University Hospitals Bristol, NHS Foundation Trust, Bristol, UK; [5]Dipartimento di Cardiologia, Universitá di Verona, Verona, Italy; [6]Great Ormond Street Hospital for Children, NHS Foundation Trust, London, UK

Corresponding author:
Dr Giovanni Biglino
Bristol Heart Institute
Bristol Royal Infirmary
Upper Maudlin Street
BS2 8HW Bristol
United Kingdom
Email: g.biglino@bristol.ac.uk
Tel: (44) 117 342 3287

For peer review only

Word count (main text including figure legends): 3,568

Total number of tables: 5

Total number of figures: 3

For peer review only - http://bmjopen.bmj.com/site/about/guidelines.xhtml

Structured Abstract (292 words)

Objective: To evaluate the feasibility of 3D printing models of coronary artery anomalies based on cardiac computed tomography (CT) data and explore their potential for clinical applications.

Design: Cardiac CT datasets of patients with various coronary artery anomalies (n=8) were retrospectively reviewed and processed, reconstructing detailed 3D models to be printed in-house with a desktop 3D printer (Form2, Formlabs) using white resin.

Setting: University Hospital, division of cardiology.

Participants: The CT scans, first, and then 3D-printed models were presented to groups of clinicians (n=8) and cardiovascular researchers (n=9).

Intervention: Participants were asked to assess different features of the 3D models and to rate the models' overall potential usefulness.

Outcome measures: Models were rated according to clarity of anatomical detail, insight into the coronary abnormality, overall perceived usefulness, and comparison to CT scans. Assessment of model characteristics used Likert-type questions (5-point scale from "Strongly disagree" to "Strongly agree") or a 10-point rating (from 0, lowest, to 10, highest). The questionnaire included a feedback form summarising overall usefulness. Participants' imaging experience (in number of years) was also recorded.

Results: All models were reconstructed and printed successfully, with accurate details showing coronary anatomy (e.g. anomalous coronary artery, coronary roofing, or coronary aneurysm in a patient with Kawasaki syndrome). All clinicians and researchers provided feedback, with both groups finding the models helpful in displaying coronary artery anatomy and abnormalities, and complementary to viewing 3D CT scans. The clinicians' group, who had substantially more imaging expertise, provided more enthusiastic ratings in terms of models' clarity, usefulness, and future use on average.

For peer review only

For peer review only - http://bmjopen.bmj.com/site/about/guidelines.xhtml

Conclusions: 3D printed heart models can be feasibly used to recreate coronary artery anatomy and enhance understanding of coronary abnormalities. Future studies can evaluate their cost-effectiveness, as well as potentially explore other printing techniques and materials.

Key Words

Coronary artery anomalies; 3D printing; Computed Tomography; Coronary arteries

Abbreviations List

3D: Three-dimensional

CT: Computed tomographyCAA: Coronary artery anomalies

ALCAPA: Anomalous left coronary artery from pulmonary artery

TGA: Transposition of the great arteries

Data Statement

Data available upon request.

For peer review only

Article Summary

Strengths and Limitations of this Study

- The study gathers evidence towards clinical and research uses of 3D cardiac models through involvement of both clinicians and researchers as key stakeholders.
- Feedback obtained from stakeholders provided qualitative and quantitative information on the models' usefulness, clarity of anatomical visualisation, and insight into the abnormality.
- Stakeholders were shown CT imaging data as well as 3D cardiac models, allowing for comparisons to be drawn on their effectiveness.
- Selected cases were chosen from a database of patients having coronary CT; a general population incidence of these anomalies is unable to be estimated.
- The study is limited by the small number of participants (n=17), and further studies looking at other anomalies may be needed to extrapolate our results to varying classifications of the anomalies.

For peer review only

Introduction

Three-dimensional (3D) printing offers unique advantages in medicine, with great potential for personalisation in the face of the wide variation of anatomy, morphology and disease across individuals. This technology has seen rapid advancement in its development and applications, with evidence beginning to emerge in support of its effectiveness and clinical value.[1] Benefit has been found in pharmaceutical and interventional research, printing of anatomical models, tissue fabrication, prosthetics and implants.[2] The use of 3D models in teaching, clinical consultations, and pre-operative planning has also been assessed.[1,3,4] The technology has seen a rapid rate of adoption in congenital cardiology, with models aiding understanding of complex anatomical structures and accurately delineating anatomical morphology to make informed management decisions.[5] Areas of positive early experience include pre-operative planning, functional flow models assessing aortic valve dysfunction, device innovation, and teaching.[6,7] Enhanced understanding of complex anatomical spatial relationships through patient-personalised 3D models could lead to improving surgical outcomes.[3]

Coronary anatomy has serious implications on cardiac premature morbidity and mortality. Congenital coronary artery anomalies (CAA) present wide variation in morphology, and are prevalent in approximately 1% of the population.[8] They are usually asymptomatic and encountered as an incidental finding during coronary angiography.[9] However, CAAs are the second most common cause of sudden cardiac death in young athletes,[10] e.g. representing the predominant (61%) identifiable cardiac abnormality in a study looking at nontraumatic sudden deaths of young healthy military recruits.[11]

Acquired, non-atherosclerotic coronary artery anomalies can be seen in Kawasaki Disease, a systemic inflammatory condition of medium sized vessels, with a predilection for the coronary arteries.[12] Coronary artery aneurysms are the most common complication of Kawasaki coronary arteritis. Coronary imaging in this population is important for serial follow up, management and

For peer review only

For peer review only - http://bmjopen.bmj.com/site/about/guidelines.xhtml

prognosis. The detection of coronary complications in Kawasaki Disease can be challenging, as it usually presents in children, who have small vessel size and increased heart rates.

Coronary computed tomography (CT) angiography is currently the most widely used non-invasive imaging modality for investigating CAA, offering detailed visualisation of the anatomy and coronary arteries with higher sensitivity than invasive coronary angiography.[13,14] A single widely accepted CAA classification scheme is lacking, despite attempts by various groups to sort them by properties including clinical symptoms, anatomical appearance, origin, and risk.[9,10,15,16] The use of CT imaging within 3D printing in medicine has been widely established.[17]This study thus aims to explore the use of 3D-printed models for viewing and investigating CAAs, evaluating the feasibility of its use both clinically and in an investigative capacity. Two key stakeholders were engaged: a) clinicians, who diagnose CAAs or are involved in management decisions, and b) non-medically qualified cardiovascular researchers, who may benefit from clearer viewing of coronary arteries for research purposes.

For peer review only

Materials and Methods

Case Selection

i) Two cardiac radiology consultants with >15 years' experience in cardiac CT (NM, MH) selected n=7 cases from our Centre's database of roughly 2000 patients having coronary CT over the last 10 years. An additional case with normal coronary anatomy was included ('control case'). The criteria for selecting the cases were: Range of CAA;

ii) Available CT coronary angiogram;

iii) Consent for research use of images.

For peer review only - http://bmjopen.bmj.com/site/about/guidelines.xhtml

Cases were selected to best represent a spectrum of CAAs at different incidences and timelines. All scans were displayed on a 512x512 matrix (Table 1). An illustrated diagram of the study design is shown in figure 1.Considering the focus of the study on printing feasibility and model evaluation, the study is not considered Clinical Research as defined in the UK Policy Framework for Health and Social Care Research, and HRA and NHS REC approval were not deemed necessary by the local R&D committee.

CT protocol

Cardiac CT scans were performed on a 320-multidetector row CT system (Toshiba Aquilion ONE) or 128-slice cardiac CT (Siemens Medical Solutions, Erlangen, Germany). Patients received 20-60 mL of iodinated contrast (calculated at 20 mg iodine/kg/second for 14-20 seconds), followed by 10-40 mL of normal saline. Scan timing by bolus tracking (Toshiba) and test bolus (Siemens) were performed with region of interest in the ascending aorta.

3D Printing

The anonymised scans were processed with commercial software (Mimics Research 19.0, Materialise NV, Leuven, Belgium) reconstructing the 3D heart model following steps of layer masking, segmentation and region growing.[18] Each scan was assessed by a medical student (ML) with the guidance of a cardiologist with 10 years in cardiac CT (SME) to decide the best way to display the CAA, purposefully removing certain structures to reveal the coronary arteries and abnormality more clearly. The decision to remove parts of the cardiovascular anatomy was made on the basis that the direct line of sight to view and follow the coronary arteries was not obstructed. Models were 1:1 in size. Whilst the accuracy of the reconstruction protocol has been previously demonstrated[18], the resulting 3D reconstruction were also verified visually by the cardiologist against the original CT dataset.

For peer review only - http://bmjopen.bmj.com/site/about/guidelines.xhtml

Models were smoothed (3-matic Research 11.0, Materialise) and exported as Stereolithography (STL) files. The STL files were imported into the 3D printing software (PreForm 2.10.3, Formlabs Inc, Somerville, MA, USA) where orientation, scaffolding, and print layout were set before printing the model. The printer (Formlabs Form 2 SLA) was available in-house. Models were all printed in white resin. Upon completion of printing, the models were submerged in propanol for 25-30 minutes, then dried in a fume cupboard for an hour. The scaffolding was removed manually. Models were then assessed for quality by a biomedical engineer with 7 years' experience of 3D printing (GB) and checked for correct anatomical representation with respect to the CT scan by the cardiologist.

Models Presentation and Feedback

A questionnaire was devised to collect feedback on the models' usefulness and clarity from two groups of stakeholders (Table 2). Cardiovascular researchers (n=9) had a background in cardiovascular science but had little or no experience of imaging. Clinicians included cardiac surgeons (n=2) and cardiologists (n=6, of which n=3 with extensive experience in cardiac imaging). Cardiologists and radiologists directly involved in patients' selection and model preparation did not take part in the survey.

A presentation was given to the researchers, where all models and their respective CT scans were shown one by one, including basic clinical information for each case (e.g. age, gender, diagnosis, medical history). Screenshots of the CT scans showing coronary anatomy were displayed, followed by introduction of the model which was then passed around and examined. A similar drop-in session was organised for the clinicians to accommodate their less flexible schedules, consisting of the same presentation and feedback questionnaire. The presentation lasted approximately 1 hour. The researcher administering the survey had no prior relationship with participants.

The questionnaire focused on assessing clarity of anatomical visualisation, insight into the abnormality, overall usefulness, and comparison to CT scans. Assessment of model characteristics used Likert-type questions on a 5-point scale (from "Strongly disagree" to "Strongly agree") or a 10-

For peer review only - http://bmjopen.bmj.com/site/about/guidelines.xhtml

point rating (from 0, lowest score, to 10, highest score). The questionnaire also included a final feedback form summarising overall usefulness and asking for participants' imaging experience (in number of years). One question regarding usefulness to future practice/applications was modified slightly to suit either research or clinical practice (i.e. usefulness "as part of your clinical practice"/"as part of your research"). Stakeholders were asked to fill in a feedback form following presentation of each case and a final feedback form.

Data analysis

Analysis of questionnaire responses was carried out in Stata (v.13, StataCorp LLC, College Station, TX, USA). Responses are presented as counts or mean±SD as appropriate. Unpaired comparisons between the two study groups (clinicians vs. researchers) were carried out using Wilcoxon rank sum test. Open-ended feedback was qualitatively analysed for dominant themes.

Patient and public involvement

The study involved key stakeholders in the technology (i.e. clinicians and researchers), according to a model of social construction of technology. In this instance, patients were not involved as beyond the specific focus of the study, despite being key stakeholders in the technology.

Results

The CT scans and models of each case are shown (Figure 2). CT scans were of adequate quality for segmentation. All models were reconstructed and printed successfully. Details of each printed model are reported (Table 3). All questionnaires were completed successfully apart from one incomplete in the researchers group.

For peer review only - http://bmjopen.bmj.com/site/about/guidelines.xhtml

Coronary artery anatomy was interpreted clearly by both groups and coronary artery anomalies were identified clearly on the models by clinicians (8.9±0.8/10) and researchers (7.4±0.3/10). The abnormality was only missed in 2% of cases overall. Clinicians tended to rate the models slightly higher than researchers on average in terms of clarity of anatomy (8.9±0.8 vs. 7.5±0.4, p=0.02), abnormality (9.0±0.7 vs. 7.5±0.5, p=0.03) and usefulness (9.1±0.7 vs. 7.7±0.5, p=0.03).

Looking at both groups combined and accounting for any incomplete answer, in 98% of cases (126/130 cases), models enhanced the awareness of coronary anatomy and abnormality. Clinicians found the models more useful over viewing the CT scans alone (4.5±0.3/5), as did researchers (4.2±0.3 /5). Almost all participants (16/17) stated that the models would be useful in any future work involving coronary artery anomalies. The dominant themes emerging from clinicians' and researchers' comments are reported in Table 4. Evaluation of model usefulness is reported in detail in Table 5.

Clinicians

All clinicians had at least moderate expertise to view and interpret scans, with 5 of them having advanced imaging expertise and working with scans daily. Generally, clinicians found the coronary arteries easy to follow and reported to easily identify the anatomy and abnormality. In 89% of all cases, they agreed that the 3D model was more effective for their understanding of the abnormality than just looking at the CT scan, with 74% stating they 'strongly agreed'. Clinicians rated the models for case 6 (Transposition of Great Arteries with abnormal circumflex artery) and case 8 (Anomalous Left Coronary Artery from Pulmonary Artery) the clearest and most effective. All clinicians agreed that models would be useful in their future work with coronary artery anomalies and suggested use for:

- Junior and senior radiologists, to demonstrate the diagnostic CT anatomy to clinicians
- Explaining conditions to patients
- Surgeons, during pre-operative planning

For peer review only - http://bmjopen.bmj.com/site/about/guidelines.xhtml

Researchers

In most cases (7/9) researchers had only basic imaging expertise or less, with 5 of them having no imaging expertise. In general, researchers could interpret the model well, but their scores of clarity of anatomy and clarity of abnormality were slightly less enthusiastic than clinicians' scores. In 97% of cases, researchers agreed that viewing the 3D model was more effective for their understanding than just looking at the CT scan, with 17% 'strongly agreeing' it was more effective. Each researcher reported that in an average of 69% of cases the models enhanced their anatomical awareness, and that the course of coronary arteries was easy to follow. Researchers gave the highest ratings to case 6 (TGA), case 7 (Kawasaki's Disease coronary aneurysm, Figure 3) and case 8 (ALCAPA, Figure 4). The open-ended feedback revealed that researchers found the models a bit more difficult to interpret, but 8/9 agreed they would be useful in any future work studying coronary artery anomalies.

Researchers interestingly remarked on the potential for clinical applications of the models and particularly mentioned:

- Surgeons in making pre-surgical decisions;
- Clinicians to make management decisions;
- Helping patients and families understand disease.

Discussion

Considering the complexity of coronary artery anomalies, patient-specific models can provide enhanced visuospatial appreciation of the defect in relation to other anatomical structures and increase the user's understanding of the anatomy, as shown in this study. Enhanced anatomical appreciation has been proven to lead to better pre-operative planning and management decisions

For peer review only

For peer review only - http://bmjopen.bmj.com/site/about/guidelines.xhtml

within a clinical setting[19]. This appreciation of relational structures is particularly vital in the clinical picture of CAAs, where evaluation of the nature, location and course to determine degree of abnormality of the CAA and risk of harm to the patient can significantly alter management decisions.[9,10,20]

3D printing has been used to reconstruct and print the coronary arterial tree[21] and it has been demonstrated that 3D printing models were effective in visualising the coronary arteries.[5] At present, only two case reports present 3D printed models for the evaluation of coronary fistulae.[22,23] In these cases, the 3D-printed model anecdotally added value in management decisions. However, no studies have been carried out to evaluate clinical use of 3D printed models for coronary arteries and CAAs.

Currently, CAAs are investigated primarily by CT angiography, which has shown to have an excellent detection rate and is fast becoming the imaging modality of choice.[13,14] Although the CT scans may adequately delineate coronary anatomy, our study showed that the complementary use of 3D printed models was useful for viewing coronary anatomy and anomalies by not only researchers but also clinicians with imaging and cardiovascular background, and was considered effective for increasing their understanding of the abnormality over simply viewing the CT scans. A randomised controlled trial by Li et al. recently drew similar conclusions in comparing CT scans vs. 3D modalities for teaching purposes. In this case, 2D CT images, monochromatic 3D virtual models and monochromatic 3D printed models were used for teaching cervical and thoracic spinal anatomy and fractures. A 10-mark post-teaching examination showed that students with teaching using 3D-virtual and 3D-printed models scored higher than students in the CT group, demonstrating efficacy of 3D learning methods over 2D.[24]

For physicians, the use of a patient-specific model is preferable to looking at CT and MRI scans as they are viewed on a 2D flat screen.[25] This indicates that 3D printed models may bring an added

For peer review only - http://bmjopen.bmj.com/site/about/guidelines.xhtml

aspect to multidisciplinary meeting discussions, perhaps due to the ability to hold and rotate the model from different angles to appreciate dimensions and spatial relationships.

In our study, reconstruction and printing of the 3D heart models required a significant amount of resources and time. The average time for reconstruction and printing was 27±1.7 hours, which may not be practical in a clinical setting. Printers with superior specifications to the Formlabs Form 2 SLA used for this study could be utilised to decrease printing times, albeit at a higher initial cost (capital investment for equipment purchase). Alternatively, changing model resolution options may reduce printing times but produce a lower quality model; such changes can be explored further to determine a clinically appropriate balance of quality versus printing speed.

We calculated the average price of production for one model to be £19 GBP ($25 USD), excluding initial printer purchasing cost. Although we obtained feedback regarding the usefulness of the models, we were unable to establish cost-effectiveness due to the lack of measurable variables such as clinical outcome. Assessment of clinical feasibility relied on feedback from a small group of researchers and clinicians, which may be prone to subjectivity. Further studies to evaluate clinical effectiveness may include measures such as impact of model on clinical outcomes and decision-making. Both clinicians and researchers found the models clear and effective in displaying coronary anatomy and the anomalies, however clinicians rated the models higher on average. This difference may be explained by the intrinsic differences between the two groups, where clinicians have a stronger background in viewing imaging and anatomical understanding. This is reflected in their self-declared measure of imaging expertise, whereas 7 out of 9 researchers had only basic imaging expertise. As a result, clinicians likely have better understanding of coronary artery course and relational structures, and may be able to better appreciate the anatomy displayed by the model. Conversely, the models were still useful for researchers who were unable to fully understand the pathology from CT scans but identified it on the models. These findings suggest that the models are

For peer review only - http://bmjopen.bmj.com/site/about/guidelines.xhtml

more effectively interpreted by users with a stronger ability to understand relevant anatomy, but still hold purposeful value for individuals with less expertise. Another possible viewing method is the 3D virtual model (e.g. 3D pdf files). Although a study by Sun et al. showed that viewing physical models produced faster and accurate responses than virtual models, they may provide a more cost-friendly alternative than 3D printed models.[26] This can be explored with comparisons to CT imaging data and physical models in future studies, specifically exploring differences in spatial understanding.Both clinicians and researchers found the models of Transposition of Great Arteries (TGA) with abnormal circumflex artery and of Anomalous Left Coronary Artery from Pulmonary Artery (ALCAPA) the most effective and clearest to understand. Interestingly, comments by three clinicians suggested that the pathology for the ALCAPA case was difficult to see on CT scans, whereas the anatomy was clearly delineated on the 3D printed models. Consideration of the complexity of the anatomical differences associated with the disease –TGA and ALCAPA being congenital coronary variations with abnormal coronary origin and course– suggest that the two conditions are more difficult to understand and follow than the pathologies of other cases which scored slightly lower. 3D printed models may potentially confer the most benefit in complex pathologies; this is consistent with the results of a study where anatomical teaching for the three most complicated regions of the body was delivered effectively with 3D modelling techniques.[27]

Segmentation of the models with removal of certain structures was to some extent an operator-dependent procedure. It was, in general, met with good response. 80% of clinicians and 62% researchers reported that it facilitated viewing the anatomy and the abnormality, whilst only 5% of clinicians and researchers stated that it made it harder to appreciate the anatomy and abnormality. Two clinicians questioned whether removing parts of the heart was the best approach, as in real life "we don't have parts removed". Although no formal review has been done of effectiveness of removing structures during segmentation process, it is a technique that has been used previously.[22,23] One clinician suggested using a more flexible material that enables structures to be bent to view the underlying coronary arteries, and there may be scope in further investigations to

For peer review only - http://bmjopen.bmj.com/site/about/guidelines.xhtml

trial more flexible materials such as TangoPlus FullCure, which has been used for preoperative surgical training.[27,28] Another option is the use of silicone models. Flexible models can be created with an injection molding technique using infusion of gel materials such as silicone and urethane into the mold.[29] A more versatile material resulting in bendable structures may allow for preservation of cardiac anatomy and structures, and manual manipulation to view the coronary arteries and anomalies.

One further suggestion by both clinicians and researchers was the use of colour to distinguish coronary arteries from other structures. The colour of certain structures can be "painted" on the digital file before printing, as demonstrated by Mcmenamin et al. who reconstructed prosections of the hand and wrist and highlighted various anatomical features using the software package 3D-Coat (Kompaniya Pilgway Studio, Ukraine). This was shown to create realistic 3D replicas in which "even small nerves and vessels could be readily distinguished".[30] The in-house printer used for our study did not support printing with multiple colours, and further studies can be conducted with higher-end printers to evaluate the use of colour in looking at coronary arteries anomalies. Awareness of intramurality of anomalous coronary arteries can be immensely useful in planning operations and choosing a suitable surgical strategy. Case 4 (myocardial bridging) was able to demonstrate intramural course which improved stakeholders' understanding from viewing the CT alone, where it may be difficult to delineate. However, although 3D modelling can recreate an intramural CAA course, it does not add to data available from CT imaging. It is hence more suitable to be used for improving understanding or planning surgical approaches, rather than confirming intramurality.

The cases for this study were selected from a hospital database, thus the overall representation of CAAs in this study may be skewed towards more symptomatically severe cases requiring medical attention. Our results may therefore not be generalisable to CAAs of all severities. However, uncomplicated cases of CAA not necessitating treatment would likely not benefit significantly from a 3D printed model. Our study was limited by the relatively small number of cases. Patients were

For peer review only - http://bmjopen.bmj.com/site/about/guidelines.xhtml

For peer review only

selected by cardiac radiology consultants to provide a range of different CAAs at different stages e.g. clarifying anomaly, incidental finding, follow-up. Although a spectrum of coronary artery anomalies was successfully produced, other conditions such as atresia and duplication were not assessed due to limited general population incidence. An appreciation of the significant haemodynamic and structural differences of the different anomalies suggests that further studies looking at other anomalies may need to be done to extrapolate our results to varying classifications of the anomalies.[16] Furthermore, it would be interesting to explore differences between sub-groups, particularly between clinicians with and without imaging expertise, whereas in this study this was limited by the small sample size of participants and the focus was comparing participants with and without medical training. Wider future use of 3D printing in coronary artery anomalies may require a more standardised protocol in production and quality assessment. Independent review of the models and accuracy to scan was done in this study, showing accurate reconstruction of the models and coronary arteries. A formal method of assessment such as Bland-Altman analysis –quantifying agreement between the dimensions of CT scans and 3D models– has been used to confirm accuracy of the 3D printed models.[31,32] These can be used in the future to verify consistency.

Conclusion

Where small discrepancies in coronary anatomy can be significant in determining prognosis and presentation, it is important that the exact anatomy should be fully understood before management decisions can be appropriately made. 3D-printed heart models can be feasibly used to recreate coronary artery anatomy and enhance understanding of the abnormality. The models could complement CT scans to display anatomy and abnormalities, and in more anatomically complex cases display structural relationships not entirely clear on the scans for users with moderate or no expertise in viewing imaging data. Future studies toward the use of 3D printing in coronary artery anomalies should investigate the best purpose of use of 3D-printed models within the spectrum of

For peer review only - http://bmjopen.bmj.com/site/about/guidelines.xhtml

coronary artery anomalies, in terms of severity and classification. There may be scope for use in pre-operative planning and decision-making, as well as teaching and clinical consultations. Cost-benefit should be determined before its role can be established alongside conventional imaging alternatives for investigating coronary artery anomalies such as CT angiography.

Conflict of interest

The authors declare there was no conflict of interest upon conduction of study.

Acknowledgements and Funding

The work is supported by a David Telling Charitable Trust equipment grant and by the British Heart Foundation. The authors also acknowledge support from the Bristol National Institute for Health Research (NIHR) Biomedical Research Centre. The views expressed in this publication are those of the authors and not necessarily those of the NHS, the National Institute for Health Research or the Department of Health.

Author Contributions

ML created the models and carried out the survey, with RB, as well as analysing survey results. SME, MH, NM, EGM, CBD and MC aided with selection of cases, clinical insight and model checking. GB conceived the study and oversaw the creation of models and the analysis. ML drafted the manuscript with GB and all authors critically contributed to revising and finalising the manuscript.

Data Sharing Statement

All data pertinent to the study are included in the manuscript, no additional unpublished data.

For peer review only - http://bmjopen.bmj.com/site/about/guidelines.xhtml

References

1. Dodziuk, H., 2016. Applications of 3D printing in healthcare. Kardiochir Torakochirurgia Pol 13, 283–293. https://doi.org/10.5114/kitp.2016.62625
2. Ventola, C.L., 2014. Medical Applications for 3D Printing: Current and Projected Uses. P T 39, 704–711.
3. Bhatla, P., Tretter, J.T., Ludomirsky, A., Argilla, M., Latson, L.A., Chakravarti, S., Barker, P.C., Yoo, S.-J., McElhinney, D.B., Wake, N., Mosca, R.S., 2017. Utility and Scope of Rapid Prototyping in Patients with Complex Muscular Ventricular Septal Defects or Double-Outlet Right Ventricle: Does it Alter Management Decisions? Pediatr Cardiol 38, 103–114. https://doi.org/10.1007/s00246-016-1489-1
4. Loke, Y.-H., Harahsheh, A.S., Krieger, A., Olivieri, L.J., 2017. Usage of 3D models of tetralogy of Fallot for medical education: impact on learning congenital heart disease. BMC Medical Education 17, 54. https://doi.org/10.1186/s12909-017-0889-0
5. Javan, R., Herrin, D., Tangestanipoor, A., 2016. Understanding Spatially Complex Segmental and Branch Anatomy Using 3D Printing: Liver, Lung, Prostate, Coronary Arteries, and Circle of Willis. Acad Radiol 23, 1183–1189. https://doi.org/10.1016/j.acra.2016.04.010
6. Bartel, T., Rivard, A., Jimenez, A., Mestres, C.A., Müller, S., 2017. Medical three-dimensional printing opens up new opportunities in cardiology and cardiac surgery. Eur. Heart J. https://doi.org/10.1093/eurheartj/ehx016
7. Vukicevic, M., Mosadegh, B., Min, J.K., Little, S.H., 2017. Cardiac 3D Printing and its Future Directions. JACC Cardiovasc Imaging 10, 171–184. https://doi.org/10.1016/j.jcmg.2016.12.001
8. Yildiz, A., Okcun, B., Peker, T., Arslan, C., Olcay, A., Bulent Vatan, M., 2010. Prevalence of Coronary Artery Anomalies in 12,457 Adult Patients Who Underwent Coronary Angiography. Clin Cardiol 33, E60–E64. https://doi.org/10.1002/clc.20588
9. Ali, M., Hanley, A., McFadden, E.P., Vaughan, C.J., 2011. Coronary artery anomalies: a practical approach to diagnosis and management. Heart Asia 3, 8–12. https://doi.org/10.1136/ha.2010.003244
10. Villa, A.D., Sammut, E., Nair, A., Rajani, R., Bonamini, R., Chiribiri, A., 2016. Coronary artery anomalies overview: The normal and the abnormal. World J Radiol 8, 537–555. https://doi.org/10.4329/wjr.v8.i6.537
11. Eckart, R.E., Scoville, S.L., Campbell, C.L., Shry, E.A., Stajduhar, K.C., Potter, R.N., Pearse, L.A., Virmani, R., 2004. Sudden death in young adults: a 25-year review of autopsies in military recruits. Ann. Intern. Med. 141, 829–834.
12. Kawasaki, T., Kosaki, F., Okawa, S., Shigematsu, I., and Yanagawa, H. A new infantile acute febrile mucocutaneous lymph node syndrome (MLNS) prevailing in Japan. Pediatrics. 1974; 54: 271–276.
13. Ghadri, J.R., Kazakauskaite, E., Braunschweig, S., Burger, I.A., Frank, M., Fiechter, M., Gebhard, C., Fuchs, T.A., Templin, C., Gaemperli, O., Lüscher, T.F., Schmied, C., Kaufmann, P.A., 2014. Congenital coronary anomalies detected by coronary computed tomography compared to invasive coronary angiography. BMC Cardiovascular Disorders 14, 81. https://doi.org/10.1186/1471-2261-14-81
14. Budoff, M.J., Ahmed, V., Gul, K.M., Mao, S.S., Gopal, A., 2006. Coronary anomalies by cardiac computed tomographic angiography. Clin Cardiol 29, 489–493. https://doi.org/10.1002/clc.4960291104
15. Dodge-Khatami, A., Mavroudis, C., Backer, C.L., 2000. Congenital Heart Surgery Nomenclature and Database Project: anomalies of the coronary arteries. Ann. Thorac. Surg. 69, S270-297.
16. Shriki, J.E., Shinbane, J.S., Rashid, M.A., Hindoyan, A., Withey, J.G., DeFrance, A., Cunningham, M., Oliveira, G.R., Warren, B.H., Wilcox, A., 2012. Identifying, characterizing,

For peer review only - http://bmjopen.bmj.com/site/about/guidelines.xhtml

and classifying congenital anomalies of the coronary arteries. Radiographics 32, 453–468. https://doi.org/10.1148/rg.322115097
17. Otton JM, Birbara NS, Hussain T, Greil G, Foley TA, Pather N. 3D printing from cardiovascular CT: a practical guide and review. Cardiovasc Diagn Ther. 2017 Oct;7(5):507–26.
18. Schievano, S., Migliavacca, F., Coats, L., Khambadkone, S., Carminati, M., Wilson, N., Deanfield, J.E., Bonhoeffer, P., Taylor, A.M., 2007. Percutaneous pulmonary valve implantation based on rapid prototyping of right ventricular outflow tract and pulmonary trunk from MR data. Radiology 242, 490–497. https://doi.org/10.1148/radiol.2422051994
19. Smith, M.L., McGuinness, J., O'Reilly, M.K., Nolke, L., Murray, J.G., Jones, J.F.X., 2017. The role of 3D printing in preoperative planning for heart transplantation in complex congenital heart disease. Ir J Med Sci. https://doi.org/10.1007/s11845-017-1564-5
20. Reul, R.M., Cooley, D.A., Hallman, G.L., Reul, G.J., 2002. Surgical Treatment of Coronary Artery Anomalies. Tex Heart Inst J 29, 299–307.
21. Hinton, T.J., Jallerat, Q., Palchesko, R.N., Park, J.H., Grodzicki, M.S., Shue, H.-J., Ramadan, M.H., Hudson, A.R., Feinberg, A.W., 2015. Three-dimensional printing of complex biological structures by freeform reversible embedding of suspended hydrogels. Science Advances 1, e1500758. https://doi.org/10.1126/sciadv.1500758
22. Velasco Forte, M.N., Byrne, N., Valverde Perez, I., Bell, A., Gómez-Ciriza, G., Krasemann, T., Sievert, H., Simpson, J., Pushparajah, K., Razavi, R., Qureshi, S., Hussain, T., 2017. 3D printed models in patients with coronary artery fistulae: anatomical assessment and interventional planning. EuroIntervention 13, e1080–e1083. https://doi.org/10.4244/EIJ-D-16-00897
23. Aroney, N., Lau, K., Daniele, L., Burstow, D., Walters, D., 2017. Three-dimensional printing: to guide management of a right coronary artery to left ventricular fistula. Eur Heart J Cardiovasc Imaging. https://doi.org/10.1093/ehjci/jex317
24. Li, Zhenzhu, Li, Zefu, Xu, R., Li, M., Li, J., Liu, Y., Sui, D., Zhang, W., Chen, Z., 2015. Three-dimensional printing models improve understanding of spinal fracture--A randomized controlled study in China. Sci Rep 5, 11570. https://doi.org/10.1038/srep11570
25. Gross, B.C., Erkal, J.L., Lockwood, S.Y., Chen, C., Spence, D.M., 2014. Evaluation of 3D printing and its potential impact on biotechnology and the chemical sciences. Anal. Chem. 86, 3240–3253. https://doi.org/10.1021/ac403397r
26. Sun L, Fukuda T, Tokuhara T, Yabuki N. Differences in spatial understanding between physical and virtual models. Frontiers of Architectural Research. 2014 Mar 1;3(1):28–35.
27. Pujol, S., Baldwin, M., Nassiri, J., Kikinis, R., Shaffer, K., 2016. Using 3D Modeling Techniques to Enhance Teaching of Difficult Anatomical Concepts. Acad Radiol 23, 507–516. https://doi.org/10.1016/j.acra.2015.12.012
28. Yoo, S.-J., Spray, T., Austin, E.H., Yun, T.-J., van Arsdell, G.S., 2017. Hands-on surgical training of congenital heart surgery using 3-dimensional print models. J. Thorac. Cardiovasc. Surg. 153, 1530–1540. https://doi.org/10.1016/j.jtcvs.2016.12.054
29. Shiraishi, I., Yamagishi, M., Hamaoka, K., Fukuzawa, M., Yagihara, T., 2010. Simulative operation on congenital heart disease using rubber-like urethane stereolithographic biomodels based on 3D datasets of multislice computed tomography. Eur J Cardiothorac Surg 37, 302–306. https://doi.org/10.1016/j.ejcts.2009.07.046
30. McMenamin, P.G., Quayle, M.R., McHenry, C.R., Adams, J.W., 2014. The production of anatomical teaching resources using three-dimensional (3D) printing technology. Anat Sci Educ 7, 479–486. https://doi.org/10.1002/ase.1475
31. Valverde, I., Gomez, G., Suarez-Mejias, C., Hosseinpour, A.-R., Hazekamp, M., Roest, A., Vazquez-Jimenez, J.F., El-Rassi, I., Uribe, S., Gomez-Cia, T., 2015. 3D printed cardiovascular models for surgical planning in complex congenital heart diseases. J Cardiovasc Magn Reson 17. https://doi.org/10.1186/1532-429X-17-S1-P196
32. Olejník, P., Nosal, M., Havran, T., Furdova, A., Cizmar, M., Slabej, M., Thurzo, A., Vitovic, P., Klvac, M., Acel, T., Masura, J., 2017. Utilisation of three-dimensional printed heart models

For peer review only - http://bmjopen.bmj.com/site/about/guidelines.xhtml

for operative planning of complex congenital heart defects. Kardiol Pol 75, 495–501. https://doi.org/10.5603/KP.a2017.0033

For peer review only

For peer review only - http://bmjopen.bmj.com/site/about/guidelines.xhtml

Table 1: Acquisition and clinical information of patients and their scans for each case.

CASES	ACQUISITION INFORMATION			CLINICAL INFORMATION		
	Scanner	Slice Thickness (mm)	Detectors	Age (y)	Gender	Pathology
**Case 1**	Toshiba Aquilion ONE	0.50	320	39	M	Normal coronary anatomy
**Case 2**	Toshiba Aquilion ONE	0.50	320	13	F	Multiple anomalous coronary arteries
**Case 3**	Toshiba Aquilion ONE	0.50	320	10	F	Coronary fistula
**Case 4**	Toshiba Aquilion ONE	0.50	320	36	M	Myocardial bridging of coronary artery
**Case 5**	SOMATOM Definition AS+	0.75	128	48	M	Tetralogy of Fallot; LCX and LAD come off separately from aorta
**Case 6**	Toshiba Aquilion ONE	0.50	320	18	M	Transposition of Great Arteries; Abnormal circumflex artery
**Case 7**	SOMATOM Definition AS+	0.75	128	52	F	Kawasaki's disease; Left main stem coronary artery aneurysm
**Case 8**	Toshiba Aquilion ONE	0.50	320	56	F	Anomalous left coronary artery from pulmonary artery (ALCAPA)

For peer review only

Table 2: Purposes for inclusion of various specialties within clinician group.

Party	Purpose for Inclusion in Feedback	How would 3D Modelling be Useful to this Party?
Researcher	Strong cardiovascular background but likely limited experience with cardiac imaging and congenital heart disease	Appreciation of anatomy across different congenital coronary artery diseases; potential use of models in future research
Cardiac surgeon	Experienced with cardiac anatomy, pathology and imaging; specific focus on potential use of models for pre-operative assessment	Understanding specific visuospatial anatomy abnormality; pre-operative planning and practice
Radiologist/ imaging expert	Extremely experienced with imaging; assessing whether 3D models improve/complement appreciation from CT alone	Visuospatial appreciation of abnormality in addition to what they see on scans
Cardiologist	Experienced with cardiac anatomy, pathology and imaging; assessing whether models can improve/complement understanding	Use for complex cases with difficult coronary anatomy; support management decisions based on measurable dimensions

For peer review only

Table 3: Details of each printed model.

Case	Reconstruction time (hrs)	Resin Volume (mL)	Layers (n)	Print Duration
Case 1	4.5	139.2	3202	22 hrs 51 min
Case 2	6	88.36	2498	14 hrs 24 min
Case 3	5	81.71	2282	12 hrs 43 min
Case 4	9	130.6	2626	29 hrs 30 min
Case 5	7	214.3	3225	33 hrs 05 min
Case 6	6.5	107.3	2683	17 hrs 45 min
Case 7	4.5	76.70	2479	13 hrs 45 min
Case 8	4	154.0	3334	25 hrs 01 min

For peer review only - http://bmjopen.bmj.com/site/about/guidelines.xhtml

Table 4: Dominant themes emerging from comments received by clinicians and researchers

**Clinicians' Comments**	**n**	**Researchers' Comments**	**n**
Enhanced visuospatial awareness using the model	5	Model was easier to understand after some guidance	4
It was useful to remove parts of structures to follow the coronary arteries	3	Removal of certain parts useful to give focus on the part of interest	2
Models would be effective for training of postgraduates and medical students	3	Use of colouring to delineate coronary arteries and abnormalities would make model more effective	2
ALCAPA (Case 8) was difficult to identify on the CT scans, but the anatomy was clear on the model	3	Understanding of anatomy improved as more models looked at	1
Use of colouring to delineate coronary arteries and abnormalities would make model more effective	2		

For peer review only

Case	Diagnosis	Clarity of Anatomy Average (out of 10)			Clarity of Abnormality Average (out of 10)			Usefulness Average (out of 10)			Effectiveness over CT Scan (from 1 = strongly disagree to 5 = strongly agree)		
		Clinicians	Researchers	p value	Clinicians	Researchers	p value	Clinicians	Researchers	p value	Clinicians	Researchers	p value
2	Multiple anomalous coronaries	8.9±1.5	6.9±1.5	0.021*	9.4±1.4	6.9±1.5	0.006*	9.2±1.4	6.7±1.6	0.009*	4.6±0.7	3.9±0.6	0.036*
3	Coronary fistula	7.5±3.0	7.0±1.1	0.520	7.9±2.7	6.5±1.8	0.108	8.0±2.8	7.1±1.1	0.142	4.1±0.8	4.0±0	0.607
4	Bridging	8.2±2.8	7.4±0.8	0.072	8.1±2.7	7.4±0.7	0.068	8.4±2.7	7.3±0.9	0.033*	4.4±1.1	4.3±0.5	0.365
5	Separate LCX and LAD from aorta	9.2±1.4	7.2±1.2	0.010*	9.2±1.4	7.0±1.8	0.010*	9.1±1.4	7.0±1.6	0.013	4.2±0.7	3.8±0.7	0.158
6	TGA, abnormal circumflex	9.5±0.7	7.5±0.9	0.002*	9.4±0.9	7.5±0.8	0.003*	9.5±0.8	7.6±0.8	0.003*	4.8±0.5	4.4±0.5	0.221
7	Kawasaki's aneurysm	9.2±1.4	8.1±1.1	0.041*	9.4±1.4	8.2±1.0	0.027*	9.2±1.4	8.4±0.9	0.055	4.6±0.7	4.4±0.5	0.346
8	ALCAPA	10.0±0	7.8±0.7	<0.001 *	9.9±0.3	7.8±1.0	<0.001*	9.9±0.3	8.1±0.6	<0.001*	5.0±0	4.3±0.5	0.005*

Table 5: Evaluation of model usefulness. LCX = ; LAD = ; TGA = transposition of the great arteries; ALCAPA = anomalous left coronary artery from pulmonary artery

For peer review only - http://bmjopen.bmj.com/site/about/guidelines.xhtml

Figure Legends

Figure 1: Illustrated diagram of study design.

Figure 2: CT Scans and models of each case, with the blue arrows pointing to the abnormality on the CT. Case 1 (1st row left 1.1-1.3): Normal coronary anatomy; axial view progressing inferiorly (1.1,1.2). Case 2 (1st row right 2.1-2.3): Multiple anomalous coronary arteries; axial view progressing inferiorly (2.1,2.2). Case 3 (2nd row left 3.1-3.3): Coronary fistula; Sagittal view progressing right (3.1,3.2). Case 4 (2nd row right 4.1-4.3): Myocardial bridging; Axial view progressing inferiorly (4.1,4.2). Case 5 (3rd row left 5.1-5.3): Separate LCX and LAD from aorta, tetralogy of Fallot; Coronal view (5.1) and Axial view (5.2). Case 6 (3rd row right 6.1-6.3): Transposition of Great Arteries (TGA) with abnormal circumflex artery; Axial view progressing inferiorly (6.1,6.2). Case 7 (4th row left 7.1-7.3): Kawasaki's disease with left main stem coronary artery aneurysm; Coronal view (7.1), Axial view (7.2). Case 8 (4th row right 8.1-8.3): Anomalous left coronary artery from pulmonary artery (ALCAPA); Axial view progressing inferiorly (8.1,8.2).

Figure 3: Detail of Kawasaki model for appreciation of coronary aneurysm (indicated by yellow arrow), two different views.

Figure 4: Detail of ALCAPA model for appreciation of coronary ostium in the pulmonary artery (A) and course of the LCA (B), as indicated by the yellow arrows.

For peer review only

For peer review only - http://bmjopen.bmj.com/site/about/guidelines.xhtml

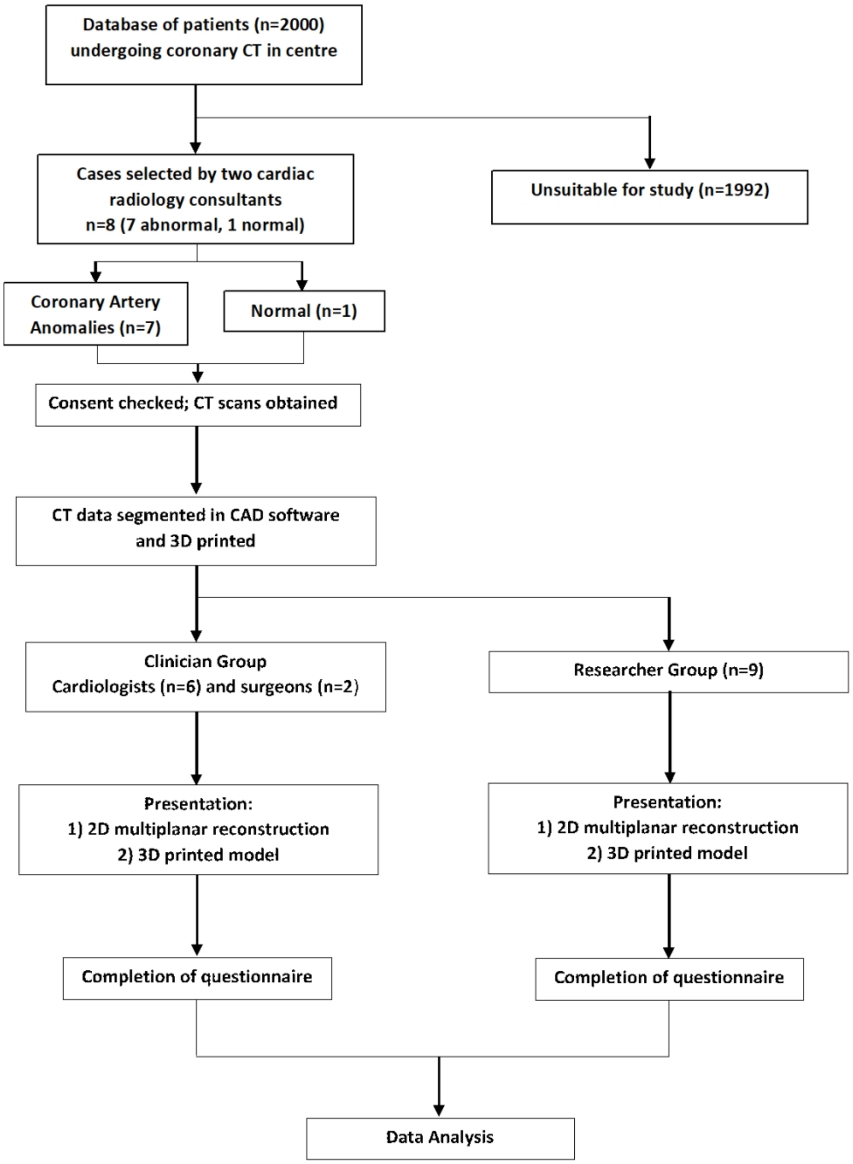
Database of patients (n=2000) undergoing coronary CT in centre
Cases selected by two cardiac radiology consultants n=8 (7 abnormal, 1 normal)
Unsuitable for study (n=1992)
Coronary Artery Anomalies (n=7)
Normal (n=1)
Consent checked; CT scans obtained
CT data segmented in CAD software and 3D printed
Clinician Group Cardiologists (n=6) and surgeons (n=2)
Researcher Group (n=9)
Presentation: 1) 2D multiplanar reconstruction 2) 3D printed model
Presentation: 1) 2D multiplanar reconstruction 2) 3D printed model
Completion of questionnaire
Completion of questionnaire
Data Analysis

Figure 1

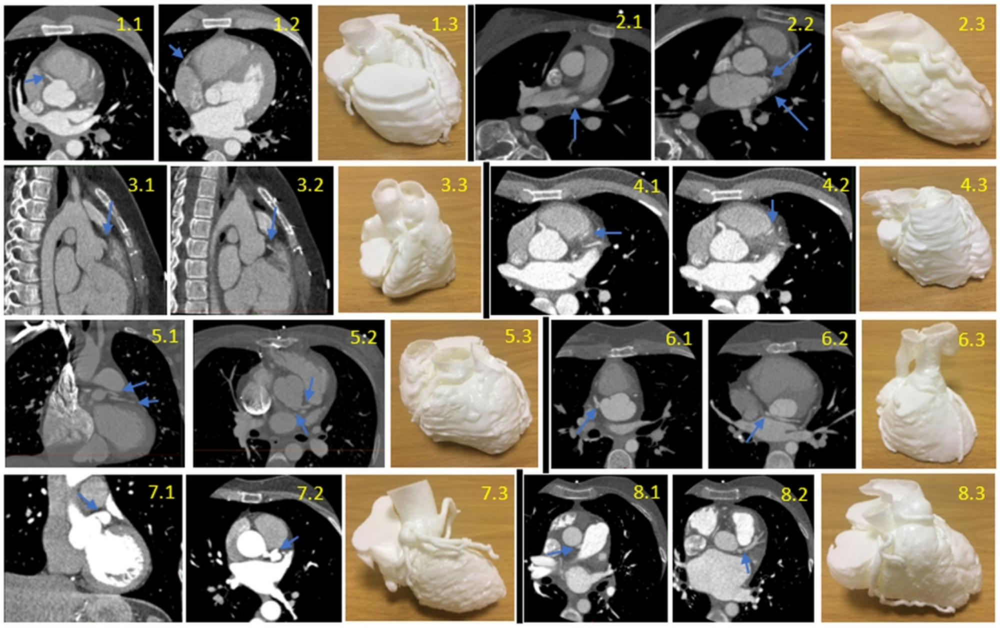

Figure 2

143x90mm (300 x 300 DPI)

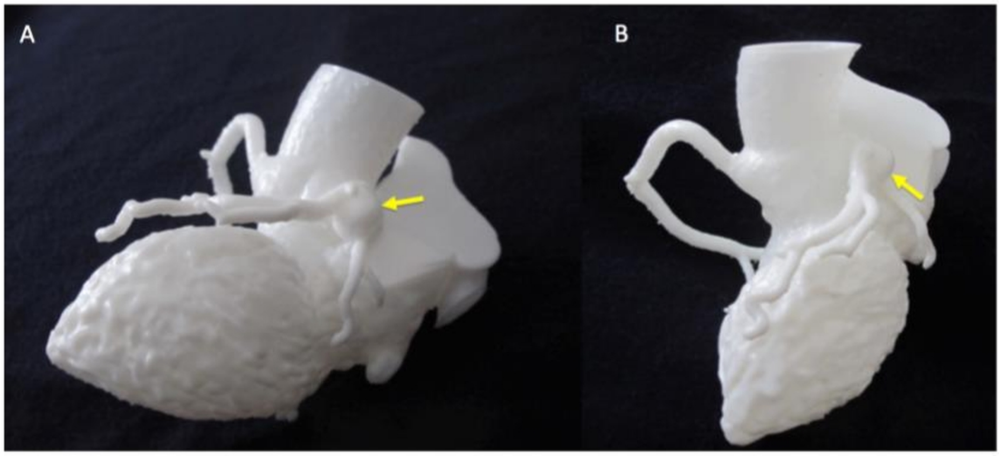

Figure 3

197x90mm (300 x 300 DPI)

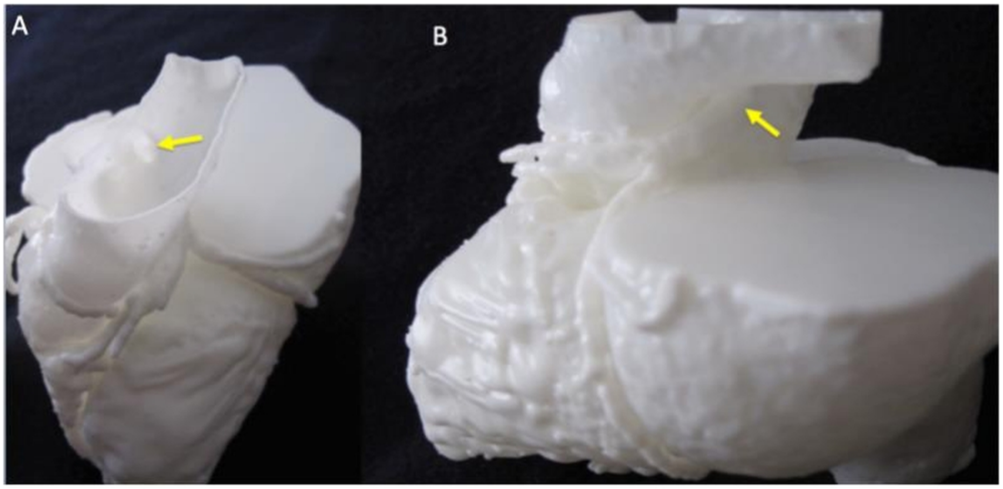

Figure 4

184x90mm (300 x 300 DPI)

Reporting checklist for qualitative study.

Based on the SRQR guidelines.

Instructions to authors

Complete this checklist by entering the page numbers from your manuscript where readers will find each of the items listed below.

Your article may not currently address all the items on the checklist. Please modify your text to include the missing information. If you are certain that an item does not apply, please write "n/a" and provide a short explanation.

Upload your completed checklist as an extra file when you submit to a journal.

In your methods section, say that you used the SRQR reporting guidelines, and cite them as:

O'Brien BC, Harris IB, Beckman TJ, Reed DA, Cook DA. Standards for reporting qualitative research: a synthesis of recommendations. Acad Med. 2014;89(9):1245-1251.

		Reporting Item	Page Number
	#1	Concise description of the nature and topic of the study identifying the study as qualitative or indicating the approach (e.g. ethnography, grounded theory) or data collection methods (e.g. interview, focus group) is recommended	1
	#2	Summary of the key elements of the study using the abstract format of the intended publication; typically includes background, purpose, methods, results and conclusions	2
Problem formulation	#3	Description and signifcance of the problem / phenomenon studied: review of relevant theory and empirical work; problem statement	5
Purpose or research question	#4	Purpose of the study and specific objectives or questions	6
Qualitative approach and research paradigm	#5	Qualitative approach (e.g. ethnography, grounded theory, case study, phenomenolgy, narrative research) and	8

		guiding theory if appropriate; identifying the research paradigm (e.g. postpositivist, constructivist / interpretivist) is also recommended; rationale. The rationale should briefly discuss the justification for choosing that theory, approach, method or technique rather than other options available; the assumptions and limitations implicit in those choices and how those choices influence study conclusions and transferability. As appropriate the rationale for several items might be discussed together.	
Researcher characteristics and reflexivity	#6	Researchers' characteristics that may influence the research, including personal attributes, qualifications / experience, relationship with participants, assumptions and / or presuppositions; potential or actual interaction between researchers' characteristics and the research questions, approach, methods, results and / or transferability	8
Context	#7	Setting / site and salient contextual factors; rationale	8
Sampling strategy	#8	How and why research participants, documents, or events were selected; criteria for deciding when no further sampling was necessary (e.g. sampling saturation); rationale	Table 2
Ethical issues pertaining to human subjects	#9	Documentation of approval by an appropriate ethics review board and participant consent, or explanation for lack thereof; other confidentiality and data security issues	6
Data collection methods	#10	Types of data collected; details of data collection procedures including (as appropriate) start and stop dates of data collection and analysis, iterative process, triangulation of sources / methods, and modification of procedures in response to evolving study findings; rationale	8
Data collection instruments and technologies	#11	Description of instruments (e.g. interview guides, questionnaires) and devices (e.g. audio recorders) used for data collection; if / how the instruments(s) changed over the course of the study	8
Units of study	#12	Number and relevant characteristics of participants, documents, or events included in the study; level of	8

For peer review only

		participation (could be reported in results)	
Data processing	#13	Methods for processing data prior to and during analysis, including transcription, data entry, data management and security, verification of data integrity, data coding, and anonymisation / deidentification of excerpts	8
Data analysis	#14	Process by which inferences, themes, etc. were identified and developed, including the researchers involved in data analysis; usually references a specific paradigm or approach; rationale	9
Techniques to enhance trustworthiness	#15	Techniques to enhance trustworthiness and credibility of data analysis (e.g. member checking, audit trail, triangulation); rationale	n/a
Syntheses and interpretation	#16	Main findings (e.g. interpretations, inferences, and themes); might include development of a theory or model, or integration with prior research or theory	9-10
Links to empirical data	#17	Evidence (e.g. quotes, field notes, text excerpts, photographs) to substantiate analytic findings	Table 4
Intergration with prior work, implications, transferability and contribution(s) to the field	#18	Short summary of main findings; explanation of how findings and conclusions connect to, support, elaborate on, or challenge conclusions of earlier scholarship; discussion of scope of application / generalizability; identification of unique contributions(s) to scholarship in a discipline or field	11-13
Limitations	#19	Trustworthiness and limitations of findings	14
Conflicts of interest	#20	Potential sources of influence of perceived influence on study conduct and conclusions; how these were managed	15
Funding	#21	Sources of funding and other support; role of funders in data collection, interpretation and reporting	15

The SRQR checklist is distributed with permission of Wolters Kluwer © 2014 by the Association of American Medical Colleges. This checklist was completed on 03. June 2018 using http://www.goodreports.org/, a tool made by the EQUATOR Network in collaboration with Penelope.ai

For peer review only

BMJ Open

Evaluating 3D printed models of coronary anomalies: a survey amongst clinicians and researchers at a University Hospital in the United Kingdom

Journal:	*BMJ Open*
Manuscript ID	bmjopen-2018-025227.R2
Article Type:	Research
Date Submitted by the Author:	09-Jan-2019
Complete List of Authors:	Lee, Matthew ; University of Bristol School of Clinical Science<br>Moharem-Elgamal, Sarah; University of Bristol, Bristol Heart Institute<br>Beckingham, Rylan; University of Bristol<br>Hamilton, Mark; University Hospitals Bristol NHS Foundation Trust<br>Manghat, Nathan; University Hospitals Bristol NHS Foundation Trust<br>Milano, Elena; Universita di Verona<br>Bucciarelli Ducci, Chiara; University of Bristol<br>Caputo, Massimo; University of Bristol, Bristol Heart Institute<br>Biglino, Giovanni; University of Bristol School of Clinical Science, Bristol Heart Institute
**Primary Subject Heading**:	Cardiovascular medicine
Secondary Subject Heading:	Communication, Radiology and imaging
Keywords:	Coronary artery anomalies, 3D printing, Computed tomography < RADIOLOGY & IMAGING, coronary arteries

SCHOLARONE™
Manuscripts

Evaluating 3D Printed Models of Coronary Anomalies: A survey Amongst Clinicians and Researchers at a University Hospital in the United Kingdom

Matthew Lee[1], Sarah Moharem-Elgamal[2,3], Rylan Beckingham[3], Mark Hamilton[4], Nathan Manghat[4], Elena Giulia Milano[3,5], Chiara Bucciarelli-Ducci[3], Massimo Caputo[3], and Giovanni Biglino[3,6]

[1]Bristol Medical School, University of Bristol, Bristol, UK ; [2]National Heart Institute, Giza, Egypt; [3]Bristol Heart Institute, University of Bristol, Bristol, UK; [4]Radiology Department, University Hospitals Bristol, NHS Foundation Trust, Bristol, UK; [5]Dipartimento di Cardiologia, Universitá di Verona, Verona, Italy; [6]Great Ormond Street Hospital for Children, NHS Foundation Trust, London, UK

Corresponding author:
Dr Giovanni Biglino
Bristol Heart Institute
Bristol Royal Infirmary
Upper Maudlin Street
BS2 8HW Bristol
United Kingdom
Email: g.biglino@bristol.ac.uk
Tel: (44) 117 342 3287

For peer review only

Word count (main text including figure legends): 3,568

Total number of tables: 5

Total number of figures: 4

Structured Abstract (296 words)

Objective: To evaluate the feasibility of 3D printing models of coronary artery anomalies based on cardiac computed tomography (CT) data and explore their potential for clinical applications.

Design: Cardiac CT datasets of patients with various coronary artery anomalies (n=8) were retrospectively reviewed and processed, reconstructing detailed 3D models to be printed in-house with a desktop 3D printer (Form2, Formlabs) using white resin.

Setting: University Hospital (division of cardiology) in the United Kingdom.

Participants: The CT scans, first, and then 3D-printed models were presented to groups of clinicians (n=8) and cardiovascular researchers (n=9).

Intervention: Participants were asked to assess different features of the 3D models and to rate the models' overall potential usefulness.

Outcome measures: Models were rated according to clarity of anatomical detail, insight into the coronary abnormality, overall perceived usefulness, and comparison to CT scans. Assessment of model characteristics used Likert-type questions (5-point scale from "Strongly disagree" to "Strongly agree") or a 10-point rating (from 0, lowest, to 10, highest). The questionnaire included a feedback form summarising overall usefulness. Participants' imaging experience (in number of years) was also recorded.

Results: All models were reconstructed and printed successfully, with accurate details showing coronary anatomy (e.g. anomalous coronary artery, coronary roofing, or coronary aneurysm in a patient with Kawasaki syndrome). All clinicians and researchers provided feedback, with both groups finding the models helpful in displaying coronary artery anatomy and abnormalities, and complementary to viewing 3D CT scans. The clinicians' group, who had substantially more imaging expertise, provided more enthusiastic ratings in terms of models' clarity, usefulness, and future use on average.

For peer review only - http://bmjopen.bmj.com/site/about/guidelines.xhtml

Conclusions: 3D printed heart models can be feasibly used to recreate coronary artery anatomy and enhance understanding of coronary abnormalities. Future studies can evaluate their cost-effectiveness, as well as potentially explore other printing techniques and materials.

Key Words

Coronary artery anomalies; 3D printing; Computed Tomography; Coronary arteries

Abbreviations List

3D: Three-dimensional

CT: Computed tomographyCAA: Coronary artery anomalies

ALCAPA: Anomalous left coronary artery from pulmonary artery

TGA: Transposition of the great arteries

Data Statement

Data available upon request.

For peer review only - http://bmjopen.bmj.com/site/about/guidelines.xhtml

Article Summary

Strengths and Limitations of this Study

- The study gathers evidence towards clinical and research uses of 3D cardiac models through involvement of both clinicians and researchers as key stakeholders.
- Feedback obtained from stakeholders provided qualitative and quantitative information on the models' usefulness, clarity of anatomical visualisation, and insight into the abnormality.
- Stakeholders were shown CT imaging data as well as 3D cardiac models, allowing for comparisons to be drawn on their effectiveness.
- Selected cases were chosen from a database of patients having coronary CT; a general population incidence of these anomalies is unable to be estimated.
- The study is limited by the small number of participants (n=17), and further studies looking at other anomalies may be needed to extrapolate our results to varying classifications of the anomalies.

For peer review only

Introduction

Three-dimensional (3D) printing offers unique advantages in medicine, with great potential for personalisation in the face of the wide variation of anatomy, morphology and disease across individuals. This technology has seen rapid advancement in its development and applications, with evidence beginning to emerge in support of its effectiveness and clinical value.[1] Benefit has been found in pharmaceutical and interventional research, printing of anatomical models, tissue fabrication, prosthetics and implants.[2] The use of 3D models in teaching, clinical consultations, and pre-operative planning has also been assessed.[1,3,4] The technology has seen a rapid rate of adoption in congenital cardiology, with models aiding understanding of complex anatomical structures and accurately delineating anatomical morphology to make informed management decisions.[5] Areas of positive early experience include pre-operative planning, functional flow models assessing aortic valve dysfunction, device innovation, and teaching.[6,7] Enhanced understanding of complex anatomical spatial relationships through patient-personalised 3D models could lead to improving surgical outcomes.[3]

Coronary anatomy has serious implications on cardiac premature morbidity and mortality. Congenital coronary artery anomalies (CAA) present wide variation in morphology, and are prevalent in approximately 1% of the population.[8] They are usually asymptomatic and encountered as an incidental finding during coronary angiography.[9] However, CAAs are the second most common cause of sudden cardiac death in young athletes,[10] e.g. representing the predominant (61%) identifiable cardiac abnormality in a study looking at nontraumatic sudden deaths of young healthy military recruits.[11]

Acquired, non-atherosclerotic coronary artery anomalies can be seen in Kawasaki Disease, a systemic inflammatory condition of medium sized vessels, with a predilection for the coronary arteries.[12] Coronary artery aneurysms are the most common complication of Kawasaki coronary arteritis. Coronary imaging in this population is important for serial follow up, management and

For peer review only

For peer review only - http://bmjopen.bmj.com/site/about/guidelines.xhtml

prognosis. The detection of coronary complications in Kawasaki Disease can be challenging, as it usually presents in children, who have small vessel size and increased heart rates.

Coronary computed tomography (CT) angiography is currently the most widely used non-invasive imaging modality for investigating CAA, offering detailed visualisation of the anatomy and coronary arteries with higher sensitivity than invasive coronary angiography.[13,14] A single widely accepted CAA classification scheme is lacking, despite attempts by various groups to sort them by properties including clinical symptoms, anatomical appearance, origin, and risk.[9,10,15,16] The use of CT imaging within 3D printing in medicine has been widely established.[17]This study thus aims to explore the use of 3D-printed models for viewing and investigating CAAs, evaluating the feasibility of its use both clinically and in an investigative capacity. Two key stakeholders were engaged: a) clinicians, who diagnose CAAs or are involved in management decisions, and b) non-medically qualified cardiovascular researchers, who may benefit from clearer viewing of coronary arteries for research purposes.

Materials and Methods

Case Selection

i) Two cardiac radiology consultants with >15 years' experience in cardiac CT (NM, MH) selected n=7 cases from our Centre's database of roughly 2000 patients having coronary CT over the last 10 years. An additional case with normal coronary anatomy was included ('control case'). The criteria for selecting the cases were: Range of CAA;

ii) Available CT coronary angiogram;

iii) Consent for research use of images.

For peer review only - http://bmjopen.bmj.com/site/about/guidelines.xhtml

Cases were selected to best represent a spectrum of CAAs at different incidences and timelines. All scans were displayed on a 512x512 matrix (Table 1). An illustrated diagram of the study design is shown in figure 1.Considering the focus of the study on printing feasibility and model evaluation, the study is not considered Clinical Research as defined in the UK Policy Framework for Health and Social Care Research, and HRA and NHS REC approval were not deemed necessary by the local R&D committee.

CT protocol

Cardiac CT scans were performed on a 320-multidetector row CT system (Toshiba Aquilion ONE) or 128-slice cardiac CT (Siemens Medical Solutions, Erlangen, Germany). Patients received 20-60 mL of iodinated contrast (calculated at 20 mg iodine/kg/second for 14-20 seconds), followed by 10-40 mL of normal saline. Scan timing by bolus tracking (Toshiba) and test bolus (Siemens) were performed with region of interest in the ascending aorta.

3D Printing

The anonymised scans were processed with commercial software (Mimics Research 19.0, Materialise NV, Leuven, Belgium) reconstructing the 3D heart model following steps of layer masking, segmentation and region growing.[18] Each scan was assessed by a medical student (ML) with the guidance of a cardiologist with 10 years in cardiac CT (SME) to decide the best way to display the CAA, purposefully removing certain structures to reveal the coronary arteries and abnormality more clearly. The decision to remove parts of the cardiovascular anatomy was made on the basis that the direct line of sight to view and follow the coronary arteries was not obstructed. Models were 1:1 in size. Whilst the accuracy of the reconstruction protocol has been previously demonstrated[18], the resulting 3D reconstruction were also verified visually by the cardiologist against the original CT dataset.

For peer review only - http://bmjopen.bmj.com/site/about/guidelines.xhtml

Models were smoothed (3-matic Research 11.0, Materialise) and exported as Stereolithography (STL) files. The STL files were imported into the 3D printing software (PreForm 2.10.3, Formlabs Inc, Somerville, MA, USA) where orientation, scaffolding, and print layout were set before printing the model. The printer (Formlabs Form 2 SLA) was available in-house. Models were all printed in white resin. Upon completion of printing, the models were submerged in propanol for 25-30 minutes, then dried in a fume cupboard for an hour. The scaffolding was removed manually. Models were then assessed for quality by a biomedical engineer with 7 years' experience of 3D printing (GB) and checked for correct anatomical representation with respect to the CT scan by the cardiologist.

Models Presentation and Feedback

A questionnaire was devised to collect feedback on the models' usefulness and clarity from two groups of stakeholders (Table 2). Cardiovascular researchers (n=9) had a background in cardiovascular science but had little or no experience of imaging. Clinicians included cardiac surgeons (n=2) and cardiologists (n=6, of which n=3 with extensive experience in cardiac imaging). Cardiologists and radiologists directly involved in patients' selection and model preparation did not take part in the survey.

A presentation was given to the researchers, where all models and their respective CT scans were shown one by one, including basic clinical information for each case (e.g. age, gender, diagnosis, medical history). Screenshots of the CT scans showing coronary anatomy were displayed, followed by introduction of the model which was then passed around and examined. A similar drop-in session was organised for the clinicians to accommodate their less flexible schedules, consisting of the same presentation and feedback questionnaire. The presentation lasted approximately 1 hour. The researcher administering the survey had no prior relationship with participants.

The questionnaire focused on assessing clarity of anatomical visualisation, insight into the abnormality, overall usefulness, and comparison to CT scans. Assessment of model characteristics used Likert-type questions on a 5-point scale (from "Strongly disagree" to "Strongly agree") or a 10-

For peer review only - http://bmjopen.bmj.com/site/about/guidelines.xhtml

point rating (from 0, lowest score, to 10, highest score). The questionnaire also included a final feedback form summarising overall usefulness and asking for participants' imaging experience (in number of years). One question regarding usefulness to future practice/applications was modified slightly to suit either research or clinical practice (i.e. usefulness "as part of your clinical practice"/"as part of your research"). Stakeholders were asked to fill in a feedback form following presentation of each case and a final feedback form.

Data analysis

Analysis of questionnaire responses was carried out in Stata (v.13, StataCorp LLC, College Station, TX, USA). Responses are presented as counts or mean±SD as appropriate. Unpaired comparisons between the two study groups (clinicians vs. researchers) were carried out using Wilcoxon rank sum test. Open-ended feedback was qualitatively analysed for dominant themes.

Patient and public involvement

The study involved key stakeholders in the technology (i.e. clinicians and researchers), according to a model of social construction of technology. In this instance, patients were not involved as beyond the specific focus of the study, despite being key stakeholders in the technology.

Results

The CT scans and models of each case are shown (Figure 2). CT scans were of adequate quality for segmentation. All models were reconstructed and printed successfully. Details of each printed model are reported (Table 3). All questionnaires were completed successfully apart from one incomplete in the researchers group.

For peer review only - http://bmjopen.bmj.com/site/about/guidelines.xhtml

For peer review only

Coronary artery anatomy was interpreted clearly by both groups and coronary artery anomalies were identified clearly on the models by clinicians (8.9±0.8/10) and researchers (7.4±0.3/10). The abnormality was only missed in 2% of cases overall. Clinicians tended to rate the models slightly higher than researchers on average in terms of clarity of anatomy (8.9±0.8 vs. 7.5±0.4, p=0.02), abnormality (9.0±0.7 vs. 7.5±0.5, p=0.03) and usefulness (9.1±0.7 vs. 7.7±0.5, p=0.03).

Looking at both groups combined and accounting for any incomplete answer, in 98% of cases (126/130 cases), models enhanced the awareness of coronary anatomy and abnormality. Clinicians found the models more useful over viewing the CT scans alone (4.5±0.3/5), as did researchers (4.2±0.3 /5). Almost all participants (16/17) stated that the models would be useful in any future work involving coronary artery anomalies. The dominant themes emerging from clinicians' and researchers' comments are reported in Table 4. Evaluation of model usefulness is reported in detail in Table 5.

Clinicians

All clinicians had at least moderate expertise to view and interpret scans, with 5 of them having advanced imaging expertise and working with scans daily. Generally, clinicians found the coronary arteries easy to follow and reported to easily identify the anatomy and abnormality. In 89% of all cases, they agreed that the 3D model was more effective for their understanding of the abnormality than just looking at the CT scan, with 74% stating they 'strongly agreed'. Clinicians rated the models for case 6 (Transposition of Great Arteries with abnormal circumflex artery) and case 8 (Anomalous Left Coronary Artery from Pulmonary Artery) the clearest and most effective. All clinicians agreed that models would be useful in their future work with coronary artery anomalies and suggested use for:

- Junior and senior radiologists, to demonstrate the diagnostic CT anatomy to clinicians
- Explaining conditions to patients

For peer review only - http://bmjopen.bmj.com/site/about/guidelines.xhtml

- Surgeons, during pre-operative planning

Researchers

In most cases (7/9) researchers had only basic imaging expertise or less, with 5 of them having no imaging expertise. In general, researchers could interpret the model well, but their scores of clarity of anatomy and clarity of abnormality were slightly less enthusiastic than clinicians' scores. In 97% of cases, researchers agreed that viewing the 3D model was more effective for their understanding than just looking at the CT scan, with 17% 'strongly agreeing' it was more effective. Each researcher reported that in an average of 69% of cases the models enhanced their anatomical awareness, and that the course of coronary arteries was easy to follow. Researchers gave the highest ratings to case 6 (TGA), case 7 (Kawasaki's Disease coronary aneurysm, Figure 3) and case 8 (ALCAPA, Figure 4). The open-ended feedback revealed that researchers found the models a bit more difficult to interpret, but 8/9 agreed they would be useful in any future work studying coronary artery anomalies.

Researchers interestingly remarked on the potential for clinical applications of the models and particularly mentioned:

- Surgeons in making pre-surgical decisions;
- Clinicians to make management decisions;
- Helping patients and families understand disease.

Discussion

Considering the complexity of coronary artery anomalies, patient-specific models can provide enhanced visuospatial appreciation of the defect in relation to other anatomical structures and

For peer review only - http://bmjopen.bmj.com/site/about/guidelines.xhtml

increase the user's understanding of the anatomy, as shown in this study. Enhanced anatomical appreciation has been proven to lead to better pre-operative planning and management decisions within a clinical setting[19]. This appreciation of relational structures is particularly vital in the clinical picture of CAAs, where evaluation of the nature, location and course to determine degree of abnormality of the CAA and risk of harm to the patient can significantly alter management decisions.[9,10,20]

3D printing has been used to reconstruct and print the coronary arterial tree[21] and it has been demonstrated that 3D printing models were effective in visualising the coronary arteries.[5] At present, only two case reports present 3D printed models for the evaluation of coronary fistulae.[22,23] In these cases, the 3D-printed model anecdotally added value in management decisions. However, no studies have been carried out to evaluate clinical use of 3D printed models for coronary arteries and CAAs.

Currently, CAAs are investigated primarily by CT angiography, which has shown to have an excellent detection rate and is fast becoming the imaging modality of choice.[13,14] Although the CT scans may adequately delineate coronary anatomy, our study showed that the complementary use of 3D printed models was useful for viewing coronary anatomy and anomalies by not only researchers but also clinicians with imaging and cardiovascular background, and was considered effective for increasing their understanding of the abnormality over simply viewing the CT scans. A randomised controlled trial by Li et al. recently drew similar conclusions in comparing CT scans vs. 3D modalities for teaching purposes. In this case, 2D CT images, monochromatic 3D virtual models and monochromatic 3D printed models were used for teaching cervical and thoracic spinal anatomy and fractures. A 10-mark post-teaching examination showed that students with teaching using 3D-virtual and 3D-printed models scored higher than students in the CT group, demonstrating efficacy of 3D learning methods over 2D.[24]

For peer review only

For physicians, the use of a patient-specific model is preferable to looking at CT and MRI scans as they are viewed on a 2D flat screen.[25] This indicates that 3D printed models may bring an added aspect to multidisciplinary meeting discussions, perhaps due to the ability to hold and rotate the model from different angles to appreciate dimensions and spatial relationships.

In our study, reconstruction and printing of the 3D heart models required a significant amount of resources and time. The average time for reconstruction and printing was 27±1.7 hours, which may not be practical in a clinical setting. Printers with superior specifications to the Formlabs Form 2 SLA used for this study could be utilised to decrease printing times, albeit at a higher initial cost (capital investment for equipment purchase). Alternatively, changing model resolution options may reduce printing times but produce a lower quality model; such changes can be explored further to determine a clinically appropriate balance of quality versus printing speed.

We calculated the average price of production for one model to be £19 GBP ($25 USD), excluding initial printer purchasing cost. Although we obtained feedback regarding the usefulness of the models, we were unable to establish cost-effectiveness due to the lack of measurable variables such as clinical outcome. Assessment of clinical feasibility relied on feedback from a small group of researchers and clinicians, which may be prone to subjectivity. Further studies to evaluate clinical effectiveness may include measures such as impact of model on clinical outcomes and decision-making. Both clinicians and researchers found the models clear and effective in displaying coronary anatomy and the anomalies, however clinicians rated the models higher on average. This difference may be explained by the intrinsic differences between the two groups, where clinicians have a stronger background in viewing imaging and anatomical understanding. This is reflected in their self-declared measure of imaging expertise, whereas 7 out of 9 researchers had only basic imaging expertise. As a result, clinicians likely have better understanding of coronary artery course and relational structures, and may be able to better appreciate the anatomy displayed by the model.

For peer review only - http://bmjopen.bmj.com/site/about/guidelines.xhtml

Conversely, the models were still useful for researchers who were unable to fully understand the pathology from CT scans but identified it on the models. These findings suggest that the models are more effectively interpreted by users with a stronger ability to understand relevant anatomy, but still hold purposeful value for individuals with less expertise. Another possible viewing method is the 3D virtual model (e.g. 3D pdf files). Although a study by Sun et al. showed that viewing physical models produced faster and accurate responses than virtual models, they may provide a more cost-friendly alternative than 3D printed models.[26] This can be explored with comparisons to CT imaging data and physical models in future studies, specifically exploring differences in spatial understanding.Both clinicians and researchers found the models of Transposition of Great Arteries (TGA) with abnormal circumflex artery and of Anomalous Left Coronary Artery from Pulmonary Artery (ALCAPA) the most effective and clearest to understand. Interestingly, comments by three clinicians suggested that the pathology for the ALCAPA case was difficult to see on CT scans, whereas the anatomy was clearly delineated on the 3D printed models. Consideration of the complexity of the anatomical differences associated with the disease –TGA and ALCAPA being congenital coronary variations with abnormal coronary origin and course– suggest that the two conditions are more difficult to understand and follow than the pathologies of other cases which scored slightly lower. 3D printed models may potentially confer the most benefit in complex pathologies; this is consistent with the results of a study where anatomical teaching for the three most complicated regions of the body was delivered effectively with 3D modelling techniques.[27]

Segmentation of the models with removal of certain structures was to some extent an operator-dependent procedure. It was, in general, met with good response. 80% of clinicians and 62% researchers reported that it facilitated viewing the anatomy and the abnormality, whilst only 5% of clinicians and researchers stated that it made it harder to appreciate the anatomy and abnormality. Two clinicians questioned whether removing parts of the heart was the best approach, as in real life "we don't have parts removed". Although no formal review has been done of effectiveness of removing structures during segmentation process, it is a technique that has been used

For peer review only - http://bmjopen.bmj.com/site/about/guidelines.xhtml

previously.[22,23] One clinician suggested using a more flexible material that enables structures to be bent to view the underlying coronary arteries, and there may be scope in further investigations to trial more flexible materials such as TangoPlus FullCure, which has been used for preoperative surgical training.[27,28] Another option is the use of silicone models. Flexible models can be created with an injection molding technique using infusion of gel materials such as silicone and urethane into the mold.[29] A more versatile material resulting in bendable structures may allow for preservation of cardiac anatomy and structures, and manual manipulation to view the coronary arteries and anomalies.

One further suggestion by both clinicians and researchers was the use of colour to distinguish coronary arteries from other structures. The colour of certain structures can be "painted" on the digital file before printing, as demonstrated by Mcmenamin et al. who reconstructed prosections of the hand and wrist and highlighted various anatomical features using the software package 3D-Coat (Kompaniya Pilgway Studio, Ukraine). This was shown to create realistic 3D replicas in which "even small nerves and vessels could be readily distinguished".[30] The in-house printer used for our study did not support printing with multiple colours, and further studies can be conducted with higher-end printers to evaluate the use of colour in looking at coronary arteries anomalies. Awareness of intramurality of anomalous coronary arteries can be immensely useful in planning operations and choosing a suitable surgical strategy. Case 4 (myocardial bridging) was able to demonstrate intramural course which improved stakeholders' understanding from viewing the CT alone, where it may be difficult to delineate. However, although 3D modelling can recreate an intramural CAA course, it does not add to data available from CT imaging. It is hence more suitable to be used for improving understanding or planning surgical approaches, rather than confirming intramurality.

The cases for this study were selected from a hospital database, thus the overall representation of CAAs in this study may be skewed towards more symptomatically severe cases requiring medical attention. Our results may therefore not be generalisable to CAAs of all severities. However,

For peer review only

uncomplicated cases of CAA not necessitating treatment would likely not benefit significantly from a 3D printed model. Our study was limited by the relatively small number of cases. Patients were selected by cardiac radiology consultants to provide a range of different CAAs at different stages e.g. clarifying anomaly, incidental finding, follow-up. Although a spectrum of coronary artery anomalies was successfully produced, other conditions such as atresia and duplication were not assessed due to limited general population incidence. An appreciation of the significant haemodynamic and structural differences of the different anomalies suggests that further studies looking at other anomalies may need to be done to extrapolate our results to varying classifications of the anomalies.[16] Furthermore, it would be interesting to explore differences between sub-groups, particularly between clinicians with and without imaging expertise, whereas in this study this was limited by the small sample size of participants and the focus was comparing participants with and without medical training. Wider future use of 3D printing in coronary artery anomalies may require a more standardised protocol in production and quality assessment. Independent review of the models and accuracy to scan was done in this study, showing accurate reconstruction of the models and coronary arteries. A formal method of assessment such as Bland-Altman analysis –quantifying agreement between the dimensions of CT scans and 3D models– has been used to confirm accuracy of the 3D printed models.[31,32] These can be used in the future to verify consistency.

Conclusion

Where small discrepancies in coronary anatomy can be significant in determining prognosis and presentation, it is important that the exact anatomy should be fully understood before management decisions can be appropriately made. 3D-printed heart models can be feasibly used to recreate coronary artery anatomy and enhance understanding of the abnormality. The models could complement CT scans to display anatomy and abnormalities, and in more anatomically complex cases display structural relationships not entirely clear on the scans for users with moderate or no

For peer review only - http://bmjopen.bmj.com/site/about/guidelines.xhtml

expertise in viewing imaging data. Future studies toward the use of 3D printing in coronary artery anomalies should investigate the best purpose of use of 3D-printed models within the spectrum of coronary artery anomalies, in terms of severity and classification. There may be scope for use in pre-operative planning and decision-making, as well as teaching and clinical consultations. Cost-benefit should be determined before its role can be established alongside conventional imaging alternatives for investigating coronary artery anomalies such as CT angiography.

Conflict of interest

The authors declare there was no conflict of interest upon conduction of study.

Acknowledgements and Funding

The work is supported by a David Telling Charitable Trust equipment grant and by the British Heart Foundation. The authors also acknowledge support from the Bristol National Institute for Health Research (NIHR) Biomedical Research Centre. The views expressed in this publication are those of the authors and not necessarily those of the NHS, the National Institute for Health Research or the Department of Health.

Author Contributions

ML created the models and carried out the survey, with RB, as well as analysing survey results. SME, MH, NM, EGM, CBD and MC aided with selection of cases, clinical insight and model checking. GB conceived the study and oversaw the creation of models and the analysis. ML drafted the manuscript with GB and all authors critically contributed to revising and finalising the manuscript.

For peer review only - http://bmjopen.bmj.com/site/about/guidelines.xhtml

For peer review only

Data Sharing Statement

All data pertinent to the study are included in the manuscript, no additional unpublished data.

For peer review only

For peer review only - http://bmjopen.bmj.com/site/about/guidelines.xhtml

References

1. Dodziuk, H., 2016. Applications of 3D printing in healthcare. Kardiochir Torakochirurgia Pol 13, 283–293. https://doi.org/10.5114/kitp.2016.62625
2. Ventola, C.L., 2014. Medical Applications for 3D Printing: Current and Projected Uses. P T 39, 704–711.
3. Bhatla, P., Tretter, J.T., Ludomirsky, A., Argilla, M., Latson, L.A., Chakravarti, S., Barker, P.C., Yoo, S.-J., McElhinney, D.B., Wake, N., Mosca, R.S., 2017. Utility and Scope of Rapid Prototyping in Patients with Complex Muscular Ventricular Septal Defects or Double-Outlet Right Ventricle: Does it Alter Management Decisions? Pediatr Cardiol 38, 103–114. https://doi.org/10.1007/s00246-016-1489-1
4. Loke, Y.-H., Harahsheh, A.S., Krieger, A., Olivieri, L.J., 2017. Usage of 3D models of tetralogy of Fallot for medical education: impact on learning congenital heart disease. BMC Medical Education 17, 54. https://doi.org/10.1186/s12909-017-0889-0
5. Javan, R., Herrin, D., Tangestanipoor, A., 2016. Understanding Spatially Complex Segmental and Branch Anatomy Using 3D Printing: Liver, Lung, Prostate, Coronary Arteries, and Circle of Willis. Acad Radiol 23, 1183–1189. https://doi.org/10.1016/j.acra.2016.04.010
6. Bartel, T., Rivard, A., Jimenez, A., Mestres, C.A., Müller, S., 2017. Medical three-dimensional printing opens up new opportunities in cardiology and cardiac surgery. Eur. Heart J. https://doi.org/10.1093/eurheartj/ehx016
7. Vukicevic, M., Mosadegh, B., Min, J.K., Little, S.H., 2017. Cardiac 3D Printing and its Future Directions. JACC Cardiovasc Imaging 10, 171–184. https://doi.org/10.1016/j.jcmg.2016.12.001
8. Yildiz, A., Okcun, B., Peker, T., Arslan, C., Olcay, A., Bulent Vatan, M., 2010. Prevalence of Coronary Artery Anomalies in 12,457 Adult Patients Who Underwent Coronary Angiography. Clin Cardiol 33, E60–E64. https://doi.org/10.1002/clc.20588
9. Ali, M., Hanley, A., McFadden, E.P., Vaughan, C.J., 2011. Coronary artery anomalies: a practical approach to diagnosis and management. Heart Asia 3, 8–12. https://doi.org/10.1136/ha.2010.003244
10. Villa, A.D., Sammut, E., Nair, A., Rajani, R., Bonamini, R., Chiribiri, A., 2016. Coronary artery anomalies overview: The normal and the abnormal. World J Radiol 8, 537–555. https://doi.org/10.4329/wjr.v8.i6.537
11. Eckart, R.E., Scoville, S.L., Campbell, C.L., Shry, E.A., Stajduhar, K.C., Potter, R.N., Pearse, L.A., Virmani, R., 2004. Sudden death in young adults: a 25-year review of autopsies in military recruits. Ann. Intern. Med. 141, 829–834.
12. Kawasaki, T., Kosaki, F., Okawa, S., Shigematsu, I., and Yanagawa, H. A new infantile acute febrile mucocutaneous lymph node syndrome (MLNS) prevailing in Japan. Pediatrics. 1974; 54: 271–276.
13. Ghadri, J.R., Kazakauskaite, E., Braunschweig, S., Burger, I.A., Frank, M., Fiechter, M., Gebhard, C., Fuchs, T.A., Templin, C., Gaemperli, O., Lüscher, T.F., Schmied, C., Kaufmann, P.A., 2014. Congenital coronary anomalies detected by coronary computed tomography compared to invasive coronary angiography. BMC Cardiovascular Disorders 14, 81. https://doi.org/10.1186/1471-2261-14-81
14. Budoff, M.J., Ahmed, V., Gul, K.M., Mao, S.S., Gopal, A., 2006. Coronary anomalies by cardiac computed tomographic angiography. Clin Cardiol 29, 489–493. https://doi.org/10.1002/clc.4960291104
15. Dodge-Khatami, A., Mavroudis, C., Backer, C.L., 2000. Congenital Heart Surgery Nomenclature and Database Project: anomalies of the coronary arteries. Ann. Thorac. Surg. 69, S270-297.
16. Shriki, J.E., Shinbane, J.S., Rashid, M.A., Hindoyan, A., Withey, J.G., DeFrance, A., Cunningham, M., Oliveira, G.R., Warren, B.H., Wilcox, A., 2012. Identifying, characterizing,

For peer review only - http://bmjopen.bmj.com/site/about/guidelines.xhtml

and classifying congenital anomalies of the coronary arteries. Radiographics 32, 453–468. https://doi.org/10.1148/rg.322115097
17. Otton JM, Birbara NS, Hussain T, Greil G, Foley TA, Pather N. 3D printing from cardiovascular CT: a practical guide and review. Cardiovasc Diagn Ther. 2017 Oct;7(5):507–26.
18. Schievano, S., Migliavacca, F., Coats, L., Khambadkone, S., Carminati, M., Wilson, N., Deanfield, J.E., Bonhoeffer, P., Taylor, A.M., 2007. Percutaneous pulmonary valve implantation based on rapid prototyping of right ventricular outflow tract and pulmonary trunk from MR data. Radiology 242, 490–497. https://doi.org/10.1148/radiol.2422051994
19. Smith, M.L., McGuinness, J., O'Reilly, M.K., Nolke, L., Murray, J.G., Jones, J.F.X., 2017. The role of 3D printing in preoperative planning for heart transplantation in complex congenital heart disease. Ir J Med Sci. https://doi.org/10.1007/s11845-017-1564-5
20. Reul, R.M., Cooley, D.A., Hallman, G.L., Reul, G.J., 2002. Surgical Treatment of Coronary Artery Anomalies. Tex Heart Inst J 29, 299–307.
21. Hinton, T.J., Jallerat, Q., Palchesko, R.N., Park, J.H., Grodzicki, M.S., Shue, H.-J., Ramadan, M.H., Hudson, A.R., Feinberg, A.W., 2015. Three-dimensional printing of complex biological structures by freeform reversible embedding of suspended hydrogels. Science Advances 1, e1500758. https://doi.org/10.1126/sciadv.1500758
22. Velasco Forte, M.N., Byrne, N., Valverde Perez, I., Bell, A., Gómez-Ciriza, G., Krasemann, T., Sievert, H., Simpson, J., Pushparajah, K., Razavi, R., Qureshi, S., Hussain, T., 2017. 3D printed models in patients with coronary artery fistulae: anatomical assessment and interventional planning. EuroIntervention 13, e1080–e1083. https://doi.org/10.4244/EIJ-D-16-00897
23. Aroney, N., Lau, K., Daniele, L., Burstow, D., Walters, D., 2017. Three-dimensional printing: to guide management of a right coronary artery to left ventricular fistula. Eur Heart J Cardiovasc Imaging. https://doi.org/10.1093/ehjci/jex317
24. Li, Zhenzhu, Li, Zefu, Xu, R., Li, M., Li, J., Liu, Y., Sui, D., Zhang, W., Chen, Z., 2015. Three-dimensional printing models improve understanding of spinal fracture--A randomized controlled study in China. Sci Rep 5, 11570. https://doi.org/10.1038/srep11570
25. Gross, B.C., Erkal, J.L., Lockwood, S.Y., Chen, C., Spence, D.M., 2014. Evaluation of 3D printing and its potential impact on biotechnology and the chemical sciences. Anal. Chem. 86, 3240–3253. https://doi.org/10.1021/ac403397r
26. Sun L, Fukuda T, Tokuhara T, Yabuki N. Differences in spatial understanding between physical and virtual models. Frontiers of Architectural Research. 2014 Mar 1;3(1):28–35.
27. Pujol, S., Baldwin, M., Nassiri, J., Kikinis, R., Shaffer, K., 2016. Using 3D Modeling Techniques to Enhance Teaching of Difficult Anatomical Concepts. Acad Radiol 23, 507–516. https://doi.org/10.1016/j.acra.2015.12.012
28. Yoo, S.-J., Spray, T., Austin, E.H., Yun, T.-J., van Arsdell, G.S., 2017. Hands-on surgical training of congenital heart surgery using 3-dimensional print models. J. Thorac. Cardiovasc. Surg. 153, 1530–1540. https://doi.org/10.1016/j.jtcvs.2016.12.054
29. Shiraishi, I., Yamagishi, M., Hamaoka, K., Fukuzawa, M., Yagihara, T., 2010. Simulative operation on congenital heart disease using rubber-like urethane stereolithographic biomodels based on 3D datasets of multislice computed tomography. Eur J Cardiothorac Surg 37, 302–306. https://doi.org/10.1016/j.ejcts.2009.07.046
30. McMenamin, P.G., Quayle, M.R., McHenry, C.R., Adams, J.W., 2014. The production of anatomical teaching resources using three-dimensional (3D) printing technology. Anat Sci Educ 7, 479–486. https://doi.org/10.1002/ase.1475
31. Valverde, I., Gomez, G., Suarez-Mejias, C., Hosseinpour, A.-R., Hazekamp, M., Roest, A., Vazquez-Jimenez, J.F., El-Rassi, I., Uribe, S., Gomez-Cia, T., 2015. 3D printed cardiovascular models for surgical planning in complex congenital heart diseases. J Cardiovasc Magn Reson 17. https://doi.org/10.1186/1532-429X-17-S1-P196
32. Olejník, P., Nosal, M., Havran, T., Furdova, A., Cizmar, M., Slabej, M., Thurzo, A., Vitovic, P., Klvac, M., Acel, T., Masura, J., 2017. Utilisation of three-dimensional printed heart models

For peer review only

for operative planning of complex congenital heart defects. Kardiol Pol 75, 495–501. https://doi.org/10.5603/KP.a2017.0033

For peer review only

Table 1: Acquisition and clinical information of patients and their scans for each case.

CASES	ACQUISITION INFORMATION			CLINICAL INFORMATION		
	Scanner	Slice Thickness (mm)	Detectors	Age (y)	Gender	Pathology
**Case 1**	Toshiba Aquilion ONE	0.50	320	39	M	Normal coronary anatomy
**Case 2**	Toshiba Aquilion ONE	0.50	320	13	F	Multiple anomalous coronary arteries
**Case 3**	Toshiba Aquilion ONE	0.50	320	10	F	Coronary fistula
**Case 4**	Toshiba Aquilion ONE	0.50	320	36	M	Myocardial bridging of coronary artery
**Case 5**	SOMATOM Definition AS+	0.75	128	48	M	Tetralogy of Fallot; LCX and LAD come off separately from aorta
**Case 6**	Toshiba Aquilion ONE	0.50	320	18	M	Transposition of Great Arteries; Abnormal circumflex artery
**Case 7**	SOMATOM Definition AS+	0.75	128	52	F	Kawasaki's disease; Left main stem coronary artery aneurysm
**Case 8**	Toshiba Aquilion ONE	0.50	320	56	F	Anomalous left coronary artery from pulmonary artery (ALCAPA)

For peer review only - http://bmjopen.bmj.com/site/about/guidelines.xhtml

Table 2: Purposes for inclusion of various specialties within clinician group.

Party	Purpose for Inclusion in Feedback	How would 3D Modelling be Useful to this Party?
Researcher	Strong cardiovascular background but likely limited experience with cardiac imaging and congenital heart disease	Appreciation of anatomy across different congenital coronary artery diseases; potential use of models in future research
Cardiac surgeon	Experienced with cardiac anatomy, pathology and imaging; specific focus on potential use of models for pre-operative assessment	Understanding specific visuospatial anatomy abnormality; pre-operative planning and practice
Radiologist/ imaging expert	Extremely experienced with imaging; assessing whether 3D models improve/complement appreciation from CT alone	Visuospatial appreciation of abnormality in addition to what they see on scans
Cardiologist	Experienced with cardiac anatomy, pathology and imaging; assessing whether models can improve/complement understanding	Use for complex cases with difficult coronary anatomy; support management decisions based on measurable dimensions

For peer review only

For peer review only - http://bmjopen.bmj.com/site/about/guidelines.xhtml

Table 3: Details of each printed model.

Case	Reconstruction time (hrs)	Resin Volume (mL)	Layers (n)	Print Duration
Case 1	4.5	139.2	3202	22 hrs 51 min
Case 2	6	88.36	2498	14 hrs 24 min
Case 3	5	81.71	2282	12 hrs 43 min
Case 4	9	130.6	2626	29 hrs 30 min
Case 5	7	214.3	3225	33 hrs 05 min
Case 6	6.5	107.3	2683	17 hrs 45 min
Case 7	4.5	76.70	2479	13 hrs 45 min
Case 8	4	154.0	3334	25 hrs 01 min

For peer review only

For peer review only - http://bmjopen.bmj.com/site/about/guidelines.xhtml

Table 4: Dominant themes emerging from comments received by clinicians and researchers

Clinicians' Comments	n	Researchers' Comments	n
Enhanced visuospatial awareness using the model	5	Model was easier to understand after some guidance	4
It was useful to remove parts of structures to follow the coronary arteries	3	Removal of certain parts useful to give focus on the part of interest	2
Models would be effective for training of postgraduates and medical students	3	Use of colouring to delineate coronary arteries and abnormalities would make model more effective	2
ALCAPA (Case 8) was difficult to identify on the CT scans, but the anatomy was clear on the model	3	Understanding of anatomy improved as more models looked at	1
Use of colouring to delineate coronary arteries and abnormalities would make model more effective	2		

For peer review only

Case	Diagnosis	Clarity of Anatomy Average (out of 10)			Clarity of Abnormality Average (out of 10)			Usefulness Average (out of 10)			Effectiveness over CT Scan (from 1 = strongly disagree to 5 = strongly agree)		
		Clinicians	Researchers	p value	Clinicians	Researchers	p value	Clinicians	Researchers	p value	Clinicians	Researchers	p value
2	Multiple anomalous coronaries	8.9±1.5	6.9±1.5	0.021*	9.4±1.4	6.9±1.5	0.006*	9.2±1.4	6.7±1.6	0.009*	4.6±0.7	3.9±0.6	0.036*
3	Coronary fistula	7.5±3.0	7.0±1.1	0.520	7.9±2.7	6.5±1.8	0.108	8.0±2.8	7.1±1.1	0.142	4.1±0.8	4.0±0	0.607
4	Bridging	8.2±2.8	7.4±0.8	0.072	8.1±2.7	7.4±0.7	0.068	8.4±2.7	7.3±0.9	0.033*	4.4±1.1	4.3±0.5	0.365
5	Separate LCX and LAD from aorta	9.2±1.4	7.2±1.2	0.010*	9.2±1.4	7.0±1.8	0.010*	9.1±1.4	7.0±1.6	0.013	4.2±0.7	3.8±0.7	0.158
6	TGA, abnormal circumflex	9.5±0.7	7.5±0.9	0.002*	9.4±0.9	7.5±0.8	0.003*	9.5±0.8	7.6±0.8	0.003*	4.8±0.5	4.4±0.5	0.221
7	Kawasaki's aneurysm	9.2±1.4	8.1±1.1	0.041*	9.4±1.4	8.2±1.0	0.027*	9.2±1.4	8.4±0.9	0.055	4.6±0.7	4.4±0.5	0.346
8	ALCAPA	10.0±0	7.8±0.7	<0.001 *	9.9±0.3	7.8±1.0	<0.001*	9.9±0.3	8.1±0.6	<0.001*	5.0±0	4.3±0.5	0.005*

Table 5: Evaluation of model usefulness. LCX = ; LAD = ; TGA = transposition of the great arteries; ALCAPA = anomalous left coronary artery from pulmonary artery

For peer review only

For peer review only - http://bmjopen.bmj.com/site/about/guidelines.xhtml

Figure Legends

Figure 1: Illustrated diagram of study design.

Figure 2: CT Scans and models of each case, with the blue arrows pointing to the abnormality on the CT. Case 1 (1st row left 1.1-1.3): Normal coronary anatomy; axial view progressing inferiorly (1.1,1.2). Case 2 (1st row right 2.1-2.3): Multiple anomalous coronary arteries; axial view progressing inferiorly (2.1,2.2). Case 3 (2nd row left 3.1-3.3): Coronary fistula; Sagittal view progressing right (3.1,3.2). Case 4 (2nd row right 4.1-4.3): Myocardial bridging; Axial view progressing inferiorly (4.1,4.2). Case 5 (3rd row left 5.1-5.3): Separate LCX and LAD from aorta, tetralogy of Fallot; Coronal view (5.1) and Axial view (5.2). Case 6 (3rd row right 6.1-6.3): Transposition of Great Arteries (TGA) with abnormal circumflex artery; Axial view progressing inferiorly (6.1,6.2). Case 7 (4th row left 7.1-7.3): Kawasaki's disease with left main stem coronary artery aneurysm; Coronal view (7.1), Axial view (7.2). Case 8 (4th row right 8.1-8.3): Anomalous left coronary artery from pulmonary artery (ALCAPA); Axial view progressing inferiorly (8.1,8.2).

Figure 3: Detail of Kawasaki model for appreciation of coronary aneurysm (indicated by yellow arrow), two different views.

Figure 4: Detail of ALCAPA model for appreciation of coronary ostium in the pulmonary artery (A) and course of the LCA (B), as indicated by the yellow arrows.

For peer review only

For peer review only - http://bmjopen.bmj.com/site/about/guidelines.xhtml

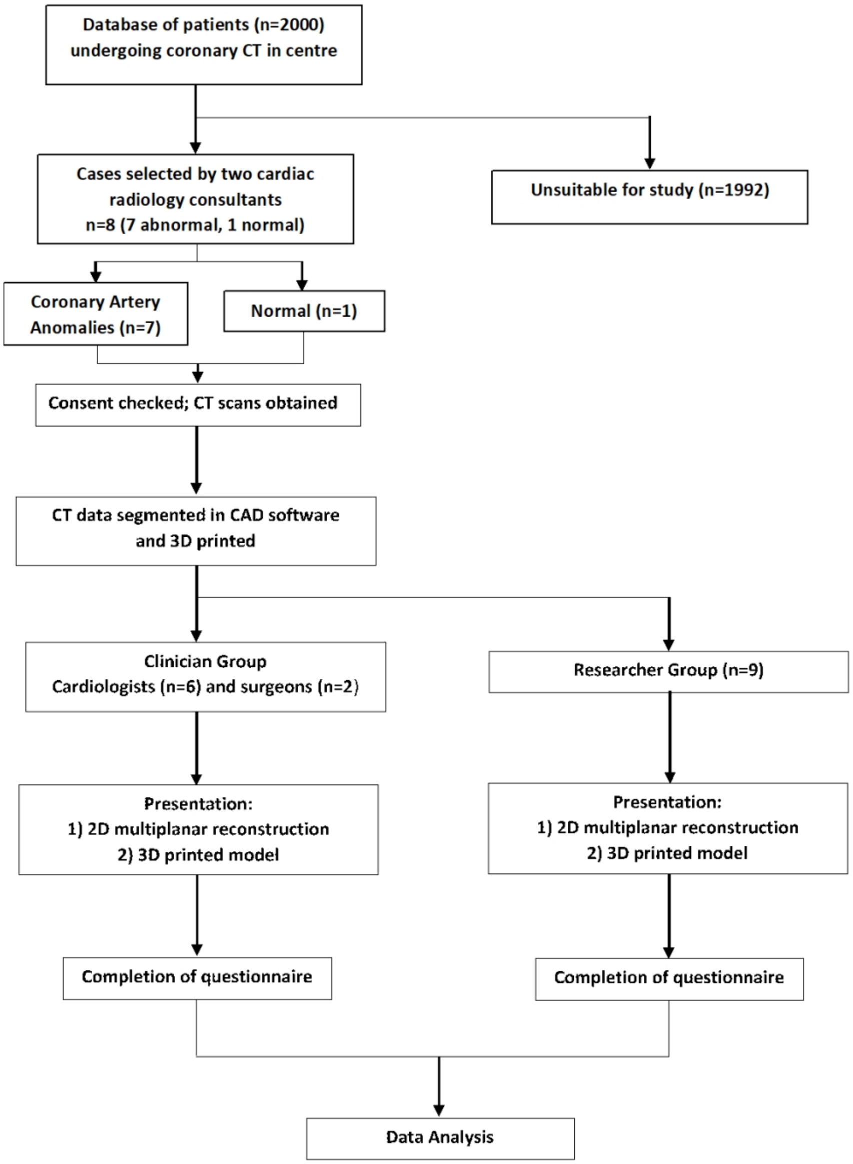
Database of patients (n=2000) undergoing coronary CT in centre
Unsuitable for study (n=1992)
Cases selected by two cardiac radiology consultants n=8 (7 abnormal, 1 normal)
Coronary Artery Anomalies (n=7)
Normal (n=1)
Consent checked; CT scans obtained
CT data segmented in CAD software and 3D printed
Clinician Group Cardiologists (n=6) and surgeons (n=2)
Researcher Group (n=9)
Presentation: 1) 2D multiplanar reconstruction 2) 3D printed model
Presentation: 1) 2D multiplanar reconstruction 2) 3D printed model
Completion of questionnaire
Completion of questionnaire
Data Analysis

Figure 1

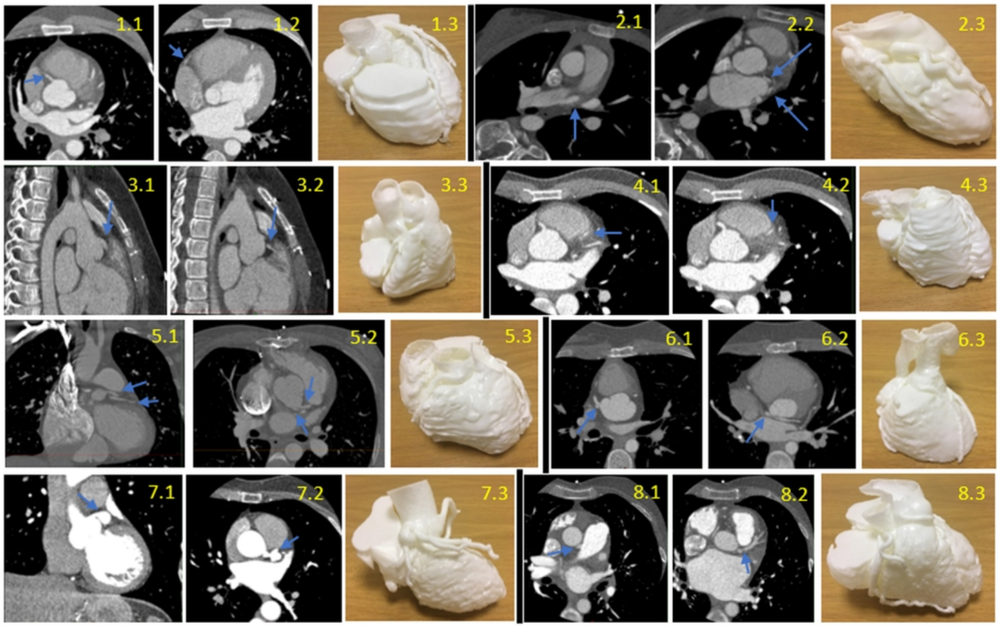

Figure 2

143x90mm (300 x 300 DPI)

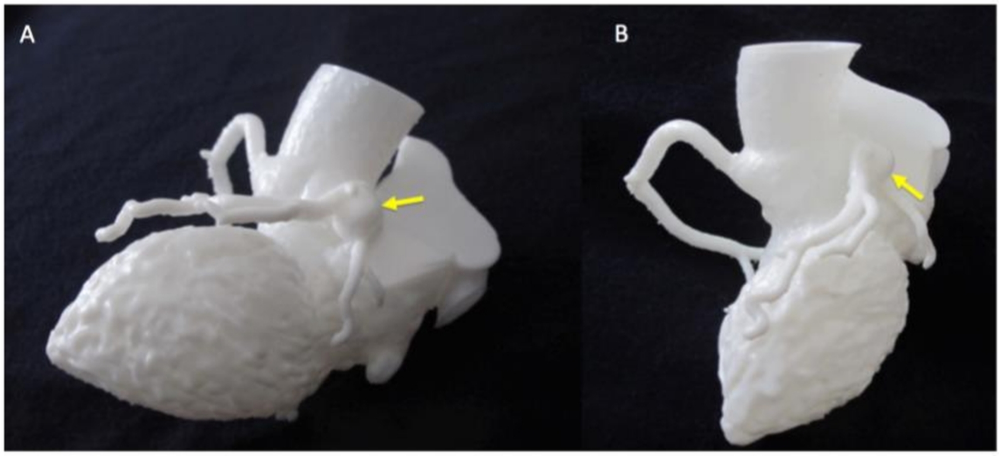

Figure 3

197x90mm (300 x 300 DPI)

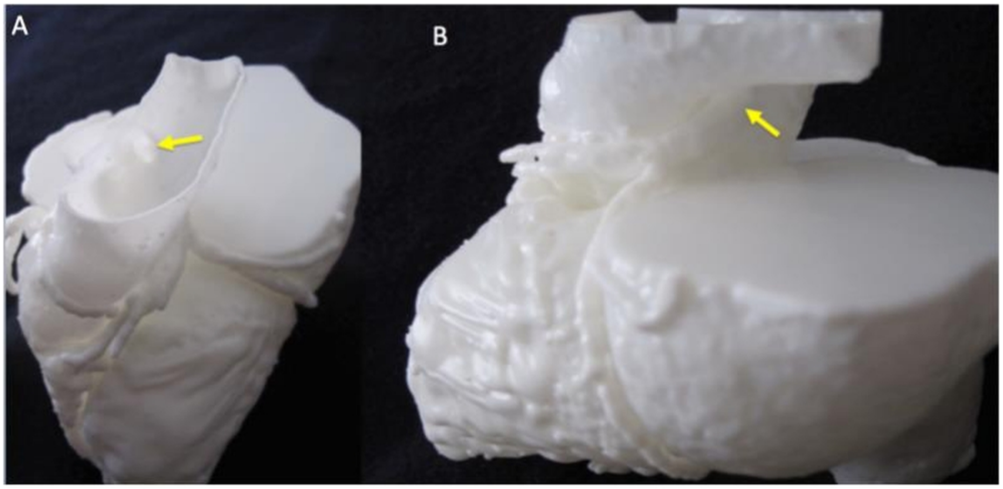

Figure 4

184x90mm (300 x 300 DPI)

Reporting checklist for qualitative study.

Based on the SRQR guidelines.

Instructions to authors

Complete this checklist by entering the page numbers from your manuscript where readers will find each of the items listed below.

Your article may not currently address all the items on the checklist. Please modify your text to include the missing information. If you are certain that an item does not apply, please write "n/a" and provide a short explanation.

Upload your completed checklist as an extra file when you submit to a journal.

In your methods section, say that you used the SRQR reporting guidelines, and cite them as:

O'Brien BC, Harris IB, Beckman TJ, Reed DA, Cook DA. Standards for reporting qualitative research: a synthesis of recommendations. Acad Med. 2014;89(9):1245-1251.

		Reporting Item	Page Number
	#1	Concise description of the nature and topic of the study identifying the study as qualitative or indicating the approach (e.g. ethnography, grounded theory) or data collection methods (e.g. interview, focus group) is recommended	1
	#2	Summary of the key elements of the study using the abstract format of the intended publication; typically includes background, purpose, methods, results and conclusions	2
Problem formulation	#3	Description and signifcance of the problem / phenomenon studied: review of relevant theory and empirical work; problem statement	5
Purpose or research question	#4	Purpose of the study and specific objectives or questions	6
Qualitative approach and research paradigm	#5	Qualitative approach (e.g. ethnography, grounded theory, case study, phenomenolgy, narrative research) and	8

For peer review only

		guiding theory if appropriate; identifying the research paradigm (e.g. postpositivist, constructivist / interpretivist) is also recommended; rationale. The rationale should briefly discuss the justification for choosing that theory, approach, method or technique rather than other options available; the assumptions and limitations implicit in those choices and how those choices influence study conclusions and transferability. As appropriate the rationale for several items might be discussed together.	
Researcher characteristics and reflexivity	#6	Researchers' characteristics that may influence the research, including personal attributes, qualifications / experience, relationship with participants, assumptions and / or presuppositions; potential or actual interaction between researchers' characteristics and the research questions, approach, methods, results and / or transferability	8
Context	#7	Setting / site and salient contextual factors; rationale	8
Sampling strategy	#8	How and why research participants, documents, or events were selected; criteria for deciding when no further sampling was necessary (e.g. sampling saturation); rationale	Table 2
Ethical issues pertaining to human subjects	#9	Documentation of approval by an appropriate ethics review board and participant consent, or explanation for lack thereof; other confidentiality and data security issues	6
Data collection methods	#10	Types of data collected; details of data collection procedures including (as appropriate) start and stop dates of data collection and analysis, iterative process, triangulation of sources / methods, and modification of procedures in response to evolving study findings; rationale	8
Data collection instruments and technologies	#11	Description of instruments (e.g. interview guides, questionnaires) and devices (e.g. audio recorders) used for data collection; if / how the instruments(s) changed over the course of the study	8
Units of study	#12	Number and relevant characteristics of participants, documents, or events included in the study; level of	8

For peer review only

		participation (could be reported in results)	
Data processing	#13	Methods for processing data prior to and during analysis, including transcription, data entry, data management and security, verification of data integrity, data coding, and anonymisation / deidentification of excerpts	8
Data analysis	#14	Process by which inferences, themes, etc. were identified and developed, including the researchers involved in data analysis; usually references a specific paradigm or approach; rationale	9
Techniques to enhance trustworthiness	#15	Techniques to enhance trustworthiness and credibility of data analysis (e.g. member checking, audit trail, triangulation); rationale	n/a
Syntheses and interpretation	#16	Main findings (e.g. interpretations, inferences, and themes); might include development of a theory or model, or integration with prior research or theory	9-10
Links to empirical data	#17	Evidence (e.g. quotes, field notes, text excerpts, photographs) to substantiate analytic findings	Table 4
Intergration with prior work, implications, transferability and contribution(s) to the field	#18	Short summary of main findings; explanation of how findings and conclusions connect to, support, elaborate on, or challenge conclusions of earlier scholarship; discussion of scope of application / generalizability; identification of unique contributions(s) to scholarship in a discipline or field	11-13
Limitations	#19	Trustworthiness and limitations of findings	14
Conflicts of interest	#20	Potential sources of influence of perceived influence on study conduct and conclusions; how these were managed	15
Funding	#21	Sources of funding and other support; role of funders in data collection, interpretation and reporting	15

The SRQR checklist is distributed with permission of Wolters Kluwer © 2014 by the Association of American Medical Colleges. This checklist was completed on 03. June 2018 using http://www.goodreports.org/, a tool made by the EQUATOR Network in collaboration with Penelope.ai

For peer review only
